# *Aspergillus fumigatus* binding IgA and IgG1 are increased in bronchoalveolar lavage fluid of horses with neutrophilic asthma

**DOI:** 10.3389/fimmu.2024.1406794

**Published:** 2024-06-17

**Authors:** Maria-Christin Jentsch, Aline Keilhaue, Bettina Wagner, Claudio Rhyner, Sabrina Lübke, Mariam Karagulyan, Corinna Arnold, Katharina L. Lohmann, Christiane L. Schnabel

**Affiliations:** ^1^ Institute of Immunology, Faculty of Veterinary Medicine, Leipzig University, Leipzig, Germany; ^2^ Department of Population Medicine and Diagnostic Sciences, College of Veterinary Medicine, Cornell University, Ithaca, NY, United States; ^3^ Christine Kühne Center for Allergy, Research, and Education (CK-CARE), Davos, Switzerland; ^4^ Swiss Institute of Allergy and Asthma Research (SIAF), Davos, Switzerland; ^5^ Department for Horses, Faculty of Veterinary Medicine, Leipzig University, Leipzig, Germany

**Keywords:** allergen, antigen, isotype, BALF, equine asthma, COPD, RAO, IAD

## Abstract

**Introduction:**

Equine asthma (EA) is a common lower airway disease in horses, but whether its pathogenesis is allergic is ambiguous. Extrinsic stimuli like hay dust induce acute exacerbation of clinical signs and sustained local neutrophilic inflammation in susceptible horses. *Aspergillus fumigatus* is an EA stimulus, but it is unclear if it merely acts as an IgE-provoking allergen. We aimed to comprehensively analyze immunoglobulin (Ig) isotypes in EA, elucidating their binding to different *A. fumigatus* antigens, and their quantities systemically in serum and locally in bronchoalveolar lavage fluid (BALF).

**Methods:**

Serum and BALF from healthy horses (HE, *n* = 18) and horses with mild-moderate asthma (MEA, *n* = 20) or severe asthma (SEA, *n* = 24) were compared. Ig isotype (IgG1, IgG3/5, IgG4/7, IgG6, IgA, and IgE) binding to nine antigens (*A. fumigatus* lysate, and recombinant Asp f 1, Asp f 7, Asp f 8, dipeptidyl-peptidase 5, class II aldolase/adducin domain protein, glucoamylase, beta-hexosaminidase, and peptide hydrolase) was compared by enzyme-linked immunosorbent assays. Total Ig isotype contents were determined by bead-based assays.

**Results:**

MEA and SEA differed from HE but hardly from each other. Compared to HE, asthmatic horses showed increased anti-*A. fumigatus* binding of IgG (BALF and serum) and IgA (BALF). Serum and BALF IgE binding and total IgE contents were similar between HE and EA. Single antigens, as well as *A. fumigatus* lysate, yielded similar Ig binding patterns. Serum and BALF IgG1 binding to all antigens was increased in SEA and to several antigens in MEA. Serum IgG4/7 binding to two antigens was increased in SEA. BALF IgA binding to all antigens was increased in SEA and MEA. Total BALF IgG1 and IgG4/7 contents were increased in SEA, and serum IgG4/7 content was increased in MEA compared to HE. Yet, total isotype contents differentiated EA and HE less clearly than antigen-binding Ig.

**Discussion:**

*A. fumigatus* immunogenicity was confirmed without identification of single dominant antigens here. *A. fumigatus* provoked elevated BALF IgG1 and IgA binding, and these isotypes appear relevant for neutrophilic EA, which does not support allergy. BALF Ig isotype differentiation beyond IgE is crucial for a comprehensive analysis of immune responses to fungi in EA pathogenesis.

## 1 Introduction

Equine asthma (EA) is a common chronic lower airway disease in horses, which has major economic impact and bears animal welfare concerns (1, 2). Affected horses show clinical signs like cough, nasal discharge, and impaired respiratory capacity, and they cannot reach full athletic performance (1–3). Two phenotypes are distinguished based on clinical signs and are described as mild to moderate equine asthma (MEA) and severe equine asthma (SEA) (1, 2). MEA includes individuals with mild clinical signs of airway disease, which can be subtle and non-specific, such as occasional cough and poor performance. Horses with MEA have normal respiratory effort at rest. This is contrary to SEA, where recurring episodes of severe clinical signs include increased respiratory effort and impaired lung function at rest (1). The severe dyspnea in SEA is caused by bronchoconstriction, increased mucus accumulation, and bronchiolar inflammation leading to obstruction (2, 4). Bronchoalveolar lavage fluid (BALF) cytology aids to further confirm the phenotypes in EA (1, 2). MEA presents with mastocytic, eosinophilic, or mild neutrophilic inflammation in BALF, while SEA is usually characterized by severe neutrophilic inflammation (1). Even though diagnostic criteria and phenotypes of EA are well defined, underlying pathological mechanisms remain incompletely understood (4, 5). To date, there is no curative treatment available, and therapy is mostly focused on hay dust avoidance and symptomatic approaches including corticosteroid application to ameliorate dyspnea (2).

Inhalation of (hay) dust triggers exacerbation of clinical signs in EA within 1 to 5 days (5–8). In addition, small airborne provoking agents such as mites, pollen, or mold have been described to cause increased clinical signs (4, 7). *Aspergillus fumigatus* (*A. fumigatus*) is a common environmental and storage fungus in hay, and was previously described as an antigen source in EA (9, 10). Airborne concentrations of *A. fumigatus* can be more than twice as high in conventional stables (straw bedding and dry hay feed) compared to stables using wood shavings bedding and pelleted feed, and husbandry in conventional stables increases the risk of EA (11–13). Respirable fractions of the mold can exceed 75% of the aerosols inside barns, which is a known health risk factor for horses and humans (12–14). *A. fumigatus* has been identified as a relevant allergen source in human asthma and allergic broncho-pulmonary aspergillosis (ABPA), and is a cause of hypersensitivity pneumonitis (alveolitis, farmer’s lung, immune complex-mediated) after long-term dust inhalation (15, 16). *A. fumigatus* protein antigens considered in EA have also been identified as allergens in human diseases [Asp f 1, Asp f 7, Asp f 8, and dipeptidyl-peptidase 5 (DPPV)], but some of these (Asp f 7 and Asp f 8) have been repeatedly suggested as antigens in analyses of EA (17–23). Additional *A. fumigatus* protein antigens that have not been described as allergens were identified by a bottom-up immunoproteomics approach analyzing binding of serum IgG from SEA (24). It is unclear if *A. fumigatus* allergens defined by human IgE reactivity are also the dominant immunogens for horses, or if there is a general dominance of single antigens in EA as implied by equine IgE binding profiles on several available allergen preparations (19, 22). Dominance of single allergens has been described in equine allergy to *Culicoides* salivary allergens, and in this context, the use of pure major allergens for serologic diagnostics is advantageous for accuracy compared to the use of mixtures like extracts (25). *A. fumigatus* lysate or extracts that are commonly used for serology contain a broad mixture of antigens without defined allergen concentrations (19, 22, 26, 27).

Asthma in humans and rodent models can be categorized into endotypes based on underlying pathogenesis mechanisms and the dominating type of immune response. T2 asthma is defined by the dominance of type 2 cytokines. In contrast, other mechanisms are central in non-T2 asthma, which includes T1 with type 1 cytokines (e.g., in hypersensitivity pneumonitis) or T3 with type 3 cytokines. In humans, T2 asthma is most common, is often IgE-mediated (allergic), and is associated with eosinophilic sputum/BALF cytology. A neutrophilic cytology, however, is mainly seen in severe, non-T2 asthma, and is often T3-associated (15).

Analyses of cellular immune responses have pointed to non-T2 asthma in SEA. Particularly increased T helper (Th) 17 cells and type 3 cytokines support T3 as an alternative endotype underlying SEA, while T2 assumptions were proposed based on increased IgE detections (4, 28–30). In MEA, underlying endotypes have not been analyzed systematically, but T2 asthma is suggested if predominant eosinophilic or mastocytic cytology is detected, in parallel to human asthma. The more diverse phenotype of MEA makes its categorization more challenging than SEA, and it is possible that several endotypes are summarized within the MEA phenotype (2, 31).

Studies aiming to analyze and identify relevant adaptive immune responses in EA yielded contradictory results (4). *A. fumigatus*-specific IgE was investigated using serum and/or BALF but showed inconclusive results with some studies indicating increased IgE in EA but a lack of this finding in others (17, 18, 26, 32–37). Additionally, IgE binding in serum differed from BALF in some studies (19, 33, 34). This suggests the analysis of both compartments and further Ig isotypes, which might elucidate mechanisms involved in EA (38–40).

Horses have 11 Ig isotypes: IgD, IgM, seven IgG subtypes (IgG1–IgG7), IgE, and IgA (38, 41). Specific monoclonal antibodies for most equine IgG subtypes have enabled their characterization. Some detect two subclasses, like IgG3/5 and IgG4/7. Increased IgG3/5 has been detected in T2 diseases with type 2 association, such as helminth infections, the allergy *Culicoides* hypersensitivity alongside IgE, and in humoral responses to soluble antigens such as tetanus toxoid vaccination (40, 42–44). IgG4/7, on the other hand, is considered type 1-associated since it is induced after viral infection, with concurrent demonstration of Th1 responses (38, 45). IgG1 is induced after viral infection, too, but also after tetanus vaccination, and in allergic responses alongside IgE, and cannot be easily associated with type 1, type 2, or type 3 immune responses (38, 43, 46, 47). According to the associations of IgE, IgG3/5, and IgG5 with type 2 and IgG4/7 with type 1 immune responses, Ig isotypes can provide some indication of underlying immune responses in horses, similar to other species (38, 48).

To the best of the authors’ knowledge, this is the first study to provide a comprehensive profile of Ig isotypes binding to *A. fumigatus* antigens systemically in serum and locally in BALF from healthy (HE), MEA, and SEA horses. We compared Ig isotype binding to *A. fumigatus* lysate and eight *A. fumigatus r* antigens as well as total Ig isotype contents between HE, MEA, and SEA.

## 2 Materials and methods

### 2.1 Preparation of *A. fumigatus* antigens for enzyme-linked immunosorbent assays

#### 2.1.1 Preparation of *A. fumigatus* lysate

*A. fumigatus* strain CBS 144.89 (CEA10), provided by Dr. Olaf Kniemeyer, Leibniz Institute for Natural Product Research and Infection Biology, Hans Knöll Institute, Jena, Germany, was grown on semi-solid growth medium (1% mycological peptone, Thermo Fisher Scientific, Waltham, MA, USA) and potassium phosphate buffer, pH 7.0 [3.4 mM KH_2_PO_4_, 5.75 mM K_2_HPO_4_, 0.06% thiamine, 2 mM MgCl_2_, 100 µg/mL chloramphenicol, gentamicin, chlortetracycline, and 20% (w/v) Kolliphor^®^ P 407, Sigma-Aldrich] at 37°C for 1–2 days, as previously described (24). *A. fumigatus* mycelium with spores was harvested, washed, and lysed by sonication in buffer {7 M urea, 2 M thiourea, and 4% 3-[(3-cholamidopropyl)-dimethylammonio]-1-propanesulfonate (CHAPS), Carl Roth, Karlsruhe, Germany}, and cleared by centrifugation and filtration, as previously described (24). The protein concentration was determined with the Bradford method using ROTI^®^Quant (Carl Roth) and albumin standard (Thermo Fisher), accounting for the buffer in the standard and blanks of the assay (49). One homogeneous batch of *A. f.* lys was frozen in aliquots at −80°C until used for enzyme-linked immunosorbent assays (ELISAs).

#### 2.1.2 Recombinant *A. fumigatus* antigens

The allergens Asp f 1, Asp f 7, and Asp f 8 of *A. fumigatus* were recombinantly expressed in *Escherichia coli* (*E. coli*) and purified as previously described (**Table 1**) (21, 24, 50–52).

**Table 1:** *A. fumigatus* lysate and *r* antigens used for ELISA.

Abbreviation	Protein name	UniProt accession number (reference)
*A. f.* lys	*Aspergillus fumigatus* (CEA10) lysate	CBS 144.89
Asp f 1 ^1^	Ribonuclease mitogillin	P67875 (50)
Asp f 7 ^1^	Allergen Asp f 7	B0Y5K9 (51, 52)
Asp f 8 ^1^	Large ribosomal subunit protein P2	B0XS47 (52)
DPPV ^1,2^	Dipeptidylpeptidase V	B0XRV0 (21, 24)
Aldo	Class II aldolase/adducin domain protein	B0XWR5 (24)
Amyl ^2^	Glucoamylase	B0XSV7 (24)
Hexo ^2^	Beta-hexosaminidase	B0Y9W3 (24)
Hydro ^2^	Peptide hydrolase	B0XX53 (24)

^1^Described as allergen (https://allergen.org), ^2^insoluble in water, processed in urea buffer.

Additional antigens were prepared as previously described (**Table 1**; **Supplementary Table 1**) (24). Briefly, coding sequences of the antigens of interest from strain *A. fumigatus* CBS 144.89 were cloned into the expression vector pET28a (+) (Sigma-Aldrich, St. Louis, MO, USA) propagated in chemically competent *E. coli* strain Rosetta pLysS (Sigma-Aldrich), cultured on lysogeny broth (LB)-agar plates containing 15 µg/mL kanamycin and 34 µg/mL chloramphenicol (Euroclone, Pero, Italy), grown overnight at 37°C, followed by a liquid sub-culture in LB medium containing the same antibiotics to prepare cryo-stocks, which were stored in 60% glycerol in LB medium at −80°C.

For antigen expression, each respective cryo-stock was cultured in 50 mL of LB medium with antibiotics overnight at 37°C and 200 rotations per minute (rpm), and then expanded 20-fold (into 1 L) under the same conditions, until OD_600_ 0.4–0.6 was reached. Isopropylthiogalactoside (IPTG, Sigma-Aldrich) was added to the culture to induce antigen expression as 6× histidine (His)-tagged antigens with individual concentrations and expression times (**Supplementary Figure 1**). Then, the bacteria were pelleted by centrifugation (5,000 × *g*, 4°C, 30 min) and the pellet was stored at −80°C for 1 to 14 days.

#### 2.1.3 *A. fumigatus r* protein purification

Bacterial pellets containing the antigens were thawed on ice by adding 5 mL of 1× phosphate buffered saline (PBS, 137 mM NaCl, 2.7 mM KCl, 13 mM Na_2_HPO_4_, and 1.5 mM KH_2_PO_4_, Carl Roth) with 200 mM NaCl, pH 7.4, protease inhibitor (Sigma-Aldrich, cOmplete™ mini, EDTA-free), lysozyme (Carl Roth), and 20 µg of DNase (Sigma-Aldrich) and were gently resuspended, brought to 20 mL with PBS, and stored on ice for 30 min. The bacterial suspensions were sonicated until no pellet was visible, lysed three times by using the French press method, and pelleted by centrifugation (48,000 × *g*, 4°C, 45 min). The first supernatant was transferred into a fresh tube and stored at 4°C, representing the soluble fraction. The pellet was dissolved by vortexing in 1 mL of urea buffer (1× PBS and 200 mM NaCl containing 8 M urea, pH 7.4), and the remaining insoluble matter was pelleted again (48,000 × *g*, 4°C, 45 min). The second supernatant was transferred into a fresh tube, representing the insoluble fraction. Both fractions’ contents of His-tagged antigens were analyzed by Western blots as described below.

Enrichment of *r* antigens within the soluble or insoluble fraction after bacterial cell lysis was detected by Coomassie Brilliant Blue G250 staining of 1D sodium dodecyl sulfate (SDS) polyacrylamide gels (**Supplementary Figure 1**). The four new antigens were predominantly detected in the insoluble fraction, and this fraction (in PBS containing 8 M urea) was used for Ni^2+-^chelate affinity chromatography on an ÄKTA™ explorer fast liquid protein chromatography (FPLC) system (Amersham Pharmacia Biotech, Freiburg, Germany) and HisTrap™ HP 1-mL columns (Cytiva, Marlborough, USA). Binding buffer [1× PBS, 200 mM NaCl, 8 M urea (Carl Roth), and 25 mM imidazole (Carl Roth), pH 7.4] and elution buffer (1× PBS, 200 mM NaCl, 8 M urea, and 500 mM imidazole, pH 7.4) were used during purification at 4°C. Aldo was found in the soluble fraction, wherefore urea was omitted in the binding buffer and the elution buffers for its purification. All antigens were eluted with a gradient of 0%–100% elution buffer over 15 column volumes. Fractions highly enriched for respective His-tagged antigens were confirmed by Western blots and were then pooled and dialyzed in 1× PBS and 200 mM NaCl (and 8 M urea for insoluble proteins), pH 7.4, at 4°C. Dialyzed samples were concentrated 10 times using Vivaspin^®^ 20 (Sartorius, Göttingen, Germany), and protein concentrations were determined using the Bradford method using ROTI^®^Quant (Carl Roth) and albumin standard (Thermo Fisher), accounting for the urea buffer in the standard and blanks of the assay (49). The samples were aliquoted and stored at **−**80°C until use for ELISA.

### 2.2 Characterization of recombinant His-tagged antigens in Western blots

SDS polyacrylamide gel electrophoresis (PAGE) was applied to determine whether antigens were contained within the soluble or insoluble fractions, and to analyze the purified antigens. For this, supernatant (20 µL), purified antigens (1µg protein per lane), or the *A. f.* lys (5 µg protein per lane, for comparison) was mixed with Laemmli buffer [0.125 M Tris-HCl, pH 6.75, 20% glycerol, 2.5% SDS, 10% 2-β-mercaptoethanol (Sigma-Aldrich), and 0.05% bromophenol blue] and boiled at 95°C for 15 min.

SDS gels [1 M Tris (Carl Roth), 0.1% SDS, pH 8.45, 0.3% ammonium persulfate (APS), and 0.03% tetramethylethylenediamine (TEMED, Merck)] containing 0.5% 2,2,2-trichloroethanol (TCE, Sigma-Aldrich) were prepared with two layers (separation 12% acrylamide, 2% bis-acrylamide, and 10% glycerol, Carl Roth) and stacking part (5.7% acrylamide and 0.6% bis-acrylamide, Carl Roth) in a Mini-PROTEAN^®^ gel casting system (Bio-Rad, Hercules, CA, USA). The same gel recipe but without TCE was applied when Coomassie Brilliant Blue G250 straining was used.

The electrophoresis was run with Tris-tricine running buffer {0.1 M Tris, 0.1 M Tricine (N-[Tris(hydroxymethyl)methyl]-glycine, Serva, Heidelberg, Germany), and 0.1% (w/v) SDS, pH 8.25, cathode} and anode buffer (0.2 M Tris, pH 8.9) in a Mini-PROTEAN Tetra Cell (Bio-Rad) with 150 W, 200 V, and constant 40 mA/gel for 1 h. Activation of TCE in SDS gels to visualize proteins by tryptophan fluorescence (TF) was achieved by 1-min exposure to 300*-*nm UV light (ECL Chemostar, iNTAS, Göttingen, Germany). All subsequent steps were performed protected from light.

Proteins from the TCE-stained SDS gel were transferred onto a nitrocellulose membrane (pore size 0.2 µm, Carl Roth) in a tank blot procedure [Mini Trans-Blot equipment (Bio-Rad), constant 300 mA, 30 min, 150 W] in transfer buffer [25 mM Tris base, 192 mM glycine (Serva), and 20% (v/v) ethanol]. Afterwards, the membranes were blocked with 1× BlueBlock PF blocking buffer (Serva) at room temperature (rt) with gentle agitation (2 rpm), for 1 h. The His-tagged *r* antigens were detected with anti-His Tag antibody (652501, BioLegend, San Diego, USA), followed by goat-anti-mouse AlexaFluor^®^ 647 (Jackson ImmunoResearch, Dianova, Hamburg, Germany) for fluorescent detection (Cy5 channel) to confirm enrichment of the correct recombinant antigens (**Supplementary Figure 1**).

### 2.3 Horse sera and BALF

#### 2.3.1 Horses, history, and physical exams for group categorization

Serum and BALF were obtained once, during diagnostic procedures from horses presented as patients to the Department for Horses, Faculty of Veterinary Medicine, Leipzig University, Leipzig, Germany, after informed consent of the owners. Additionally, samples from horses under the animal experiment permission number TVV22/20 (file number 25–5131/490/23, Landesdirektion Sachsen, Germany) were acquired once per horse, by the same procedures. Horses were carefully examined, scored, and, after completion of sample storage and data acquisition, retrospectively categorized into three phenotypic groups of healthy (HE) horses, horses suffering from MEA, or horses with SEA. In total, 62 adult horses were included (**Table 2**). Assessment consisted of history acquired during narrative anamnesis that covered previous clinical signs, husbandry, and environmental risk factors for EA (**Supplementary Table 2**) and by HOARSI [horse owner assessed respiratory signs index (53–55)], clinical signs at examination (2, 56, 57), tracheal mucus score (6), BALF cytology (1, 2), and arterial blood gas analysis as previously described (28). If HOARSI was not assessed, horses were still included if the narrative anamnesis covered chronic signs of respiratory disease as assessed by HOARSI for the time when the clinical signs were worst as noted by the owner (54, 55). Horses treated with bronchodilators or corticosteroids within 4 weeks prior to examination, according to their owner’s information, or with systemic diseases indicated by history or physical exams, were excluded from the study.

**Table 2:** Horse characteristics as medians per group (ranges).

Group characteristic	Healthy (HE)	Mild-moderate equine asthma (MEA)	Severe equine asthma (SEA)	Normal range, (total range)
*n*	18	20	24	
Age ^a^ (years)	11 (6–21)	11 (3–19)	15 (8–22)	
HOARSI ^1,m,s,a^	1 (1–2)	2 (1–4)	4 (2–4)	1 (1–4)
Clinical score ^2,s,a^	2 (0–8)	2,5 (0–6)	7 (2–17)	<3 (0–19)
Mucus score ^3,s,a^	1 (0–4)	2 (0–4)	4 (1–4)	<3 (0–5)
BALF neutrophils ^4, m,s,a^ (%)	1.6 (0.2–3.6)	7.7 (1.2–19.8)	18.8 (6.7–86.8)	<5
BALF eosinophils ^4^ (%)	0 (0–0.4)	0 (0–6)	0 (0–2.5)	<0.5
BALF mast cells ^5^ (%)	0.7 (0–3.5)	0.6 (0–4)	1.0 (0–3.2)	<5
aaΔO2 ^6,a^ (kPa)	0 (0–0.79)	0 (0–1.84)	0.54 (0–3.48)	<1.0

Statistical comparisons by Mann–Whitney tests (p > 0.05): ^m^different between HE and MEA, ^s^different between HE and SEA, ^a^different between MEA and SEA.

^1^HOARSI (horse owner assessed respiratory signs index) (53–55) was assessed in 55/62 horses; the remaining horses’ history was based on narrative anamnesis.

^2^Clinical signs as main criteria for group classification: Sum of scores for nasal discharge, nasal flare, cough, respiratory rate, abdominal lift, auscultation at rest, and after rebreathing (28, 56–58); one HE horse had an elevated score based on malformation of its upper airways and resulting clinical signs.

^3^Endoscopic tracheal mucus assessment according to Gerber et al. (6).

^4^At least 500 cells were differentiated on DiffQuick-stained cytospins.

^5^At least 500 cells were differentiated on Toluidine blue-stained cytospins.

^6^aaΔO2 arterio-alveolar partial pressure difference calculated from arterial blood gas analysis was assessed in 52/62 horses.

Clinical signs were the main criteria for categorization (**Table 2**). Horses without a history of asthma signs and without clinical signs of EA at the time of the examination were included as HE if they also had normal BAL cytology. Horses with a history or clinical signs of asthma upon examination were categorized as MEA if they lacked dyspnea at rest and severe neutrophilic inflammation (>20% neutrophils). Horses with a history of severe signs (dyspnea and coughing fits) or severe clinical signs including dyspnea at rest and abnormal neutrophilic BALF cytology were included in the SEA group.

Clinical signs were assessed by an equine internal medicine specialist who was usually aware of the horses’ history. They reported clinical signs using a detailed clinical score summarizing mucous nasal discharge, nostril flaring, cough, respiratory rate, abdominal lift, and auscultation findings as previously described (**Supplementary Table 3**) (28). This score was adapted from the 23-point score by Lavoie et al., which was designed to differentiate HE and SEA horses (56). The score applied here additionally included auscultation findings during a re-breathing exam, which has been deemed useful to identify MEA and SEA cases (57, 58).

#### 2.3.2 Endoscopy and sampling—serum and BALF

Blood was drawn from the jugular vein using a vacutainer system (Becton Dickinson GmbH, Heidelberg, Germany). The blood was allowed to clot at rt for 6 h and then at 4°C overnight. The following day, serum was separated by centrifugation (1,800 × *g*, 4°C, 10 min) and stored in aliquots at **−**80°C.

Horses were then sedated with detomidine and butorphanol and endoscopy of the upper and lower respiratory tract was performed using a flexible video endoscope (G28–250, 10.4 mm diameter, Storz, Tuttlingen, Germany), as previously described (28). Tracheal mucus was scored by the examining equine internal medicine specialist on site as described by Gerber et al. (6). After topical anesthesia of the larynx and bronchi, bronchoalveolar lavage was performed via biopsy channel of the endoscope (10.4 mm diameter) or a BAL probe (10 mm diameter plus cuff) inserted into the lung instilling 60 mL of saline per 100 kg of body weight in one bolus followed by immediate aspiration by syringes, as previously described (28).

The recovered BALF was pooled into sterile glass bottles and kept on ice for a maximum of 3 h before processing by a technician blinded to the history and clinical characteristics of the horses. BALF was passed over a cell strainer (100 µm pore size, LABSOLUTE^®^, Th. Geyer, Renningen, Germany) and centrifuged (500 × *g*, 8 min, 4°C), and cell-free BALF was frozen in aliquots at **−**80°C. BALF cytology was assessed on cytospins stained with DiffQuick (RAL Diagnostics, Martillac, France) and Toluidine blue (0.25 mg/mL Toluidine Blue O, Sigma-Aldrich, Merck KGaA, Darmstadt Germany) differentiating a minimum of 500 leukocytes, as previously described (1, 28). A maximum of 5% neutrophils, 5% mast cells, and 1% eosinophils were considered normal (1).

Serum and BALF samples were collected between November 2019 and January 2022 and stored at **−**80°C in aliquots until used in the experiments between October 2022 and February 2024. During each experimental series, samples were kept at 4°C for no longer than 3 weeks. All available samples from suitable horses that met the inclusion criteria were selected and blinded with randomized sample IDs throughout all experiments, until statistical data analysis.

### 2.4 Quantification of antigen-binding antibodies by ELISA

ELISA plates (Nunc Maxisorp flat bottom plates, Thermo Fisher) were coated with 4 µg/mL of *A. fumigatus* antigens (*A. f.* lys, Asp f 1, Asp f 7, Asp f 8, DPPV, Aldo, Amyl, Hexo, or Hydro, **Table 1**) in sodium carbonate buffer (1 M NaHCO_3_ and 1 M Na_2_CO_3_) at 4°C overnight. The plates were blocked with PBS containing 0.5% w/v BSA and 0.1% v/v gelatin (all reagents from Carl Roth) at rt for 1 h. Plates were then washed three times with PBST (PBS, 0.05% v/v Tween20, Carl Roth). Test sera were diluted in serum diluent (PBST containing 0.5% w/v BSA and 0.1% v/v gelatin, all reagents from Carl Roth), while BALF was used undiluted or diluted in PBST. Sera or BALF in assay-optimized dilutions and blanks (serum diluent for serum assays, 0.9% w/v sodium chloride for BALF) was applied to the plates and incubated for 1 h at rt (**Table 3**). After washing in PBST, incubation with primary isotype-specific detection antibodies or Peroxidase AffiniPure Goat Anti-Horse IgG (H+L) (Jackson ImmunoResearch) for Pan-Ig detection was performed for 1 h at rt (**Table 3**). Plates were washed again in PBST, followed by incubation with Peroxidase-conjugated secondary antibody [Peroxidase-conjugated AffiniPure Goat Anti-Mouse (H+L) (Jackson ImmunoResearch), 0.16 µg/mL in PBST] and washed again in PBST. Then, 3,3',5,5'-tetramethylbenzidine substrate (TMB, KPL, medac, Wedel, Germany) was added and developed at rt, in the dark, for 5–15 min (assay-optimized). The reaction was stopped with 1 M phosphoric acid (Carl Roth). Quantification of optical densities (ODs) was performed at 450 nm on a SpectraMax 340 reader with SoftMax Pro software (Molecular Devices, Thermo Fisher). Blank-reduced ODs were reported and compared.

**Table 3:** Optimized conditions for antigen-specific serum Ig quantification (ELISA).

Ig isotypes	Detection antibody (det. Ab)	Det. Ab (µg/mL)	Dilution serum ^H^	Dilution BALF ^H^
Pan-Ig	Polyclonal Peroxidase AffiniPure Goat Anti-Horse IgG (H+L) ^A^	0.4	1,500 (*A. f.* lys, Asp f 1) 300 (Asp f 7, Asp f 8) 1,000 (DPPV, Aldo, Amyl, Hexo, and Hydro)	2
IgM	Clone 1–22 ^B^	0.4 ^G^	125 (Asp f 7, Asp f 8) 500 (other antigens)	n. a. ^I^
IgG1	Clone CVS45 ^C,D^	2	250	Undiluted
IgG3/5	Clone 586 ^D^	2	125	Undiluted
IgG4/7	Clone CVS39 ^C,D^	1	250	Undiluted
IgG6	Clone 267 ^D^	1	62.5	Undiluted
IgE	Clones 134 ^E^ and 176 ^E^ (combined)	0.8 (each)	3	Undiluted
IgA	Clone BVS2 ^F^	1 ^G^	15.625	2

^A^Jackson ImmunoResearch #108–035-003; ^B^(59), ^C^(60, 61), ^D^(62), ^E^(63), ^F^(64, 65).

^G^All det. Ab were diluted in PBST, except for a-IgM and a-IgA, which were diluted in SD.

^H^To establish assay-optimized serum dilutions, at least five sera were used in at least five twofold dilutions for detection of each Ig isotype on each antigen. A dilution in the linear dynamic range of each assay for most test samples was chosen for the analysis.

^I^IgM binding in BALF was not analyzed (n. a.) due to undetectable or very low total IgM contents in BALF.

### 2.5 Total immunoglobulin isotype contents in BALF and serum

Total contents of Ig isotypes were quantified by bead-based assays as previously described for serum (62, 64, 66). Briefly, IgM, IgG1, IgG3/5, IgG4/7, and IgG6 were quantified in a multiplexed assay in undiluted BALF or 1:50,000 diluted serum, with isotype-specific monoclonal antibodies coupled to different beads (compare detection antibodies in **Table 3**) and detection by polyclonal biotinylated goat-anti-horse-Ig (H+L) (62). IgA and IgE were quantified in undiluted BALF or 1:10 diluted serum using pairs of monoclonal antibodies coupled to the beads and biotinylated for detection (64, 66). After incubation with R-phycoerythrin-conjugated streptavidin (Invitrogen, Carlsbad, CA, USA), the total content of each isotype was quantified as median fluorescent intensity (MFI) on a BioPlex200 instrument (Bio-Rad).

### 2.6 Statistical analyses

Ig binding to *A. f*. lys and antigens (blank-reduced ODs) on ELISA and the contents of total Ig isotypes (background-reduced MFI) were compared in BALF and sera between asthmatic and healthy horses by Mann–Whitney *U* tests with correction for multiple comparisons by the Holm–Šídák method (alpha = 0.05). Correlations between several parameters (Ig binding and total Ig contents) were analyzed over data from all individuals (*n* = 62) without separation into groups, using nonparametric Spearman correlation analyses with *p >* 0.05.

## 3 Results

### 3.1 Serum IgG1 binding to *A. fumigatus* antigens is elevated in EA

**Figure 1:**
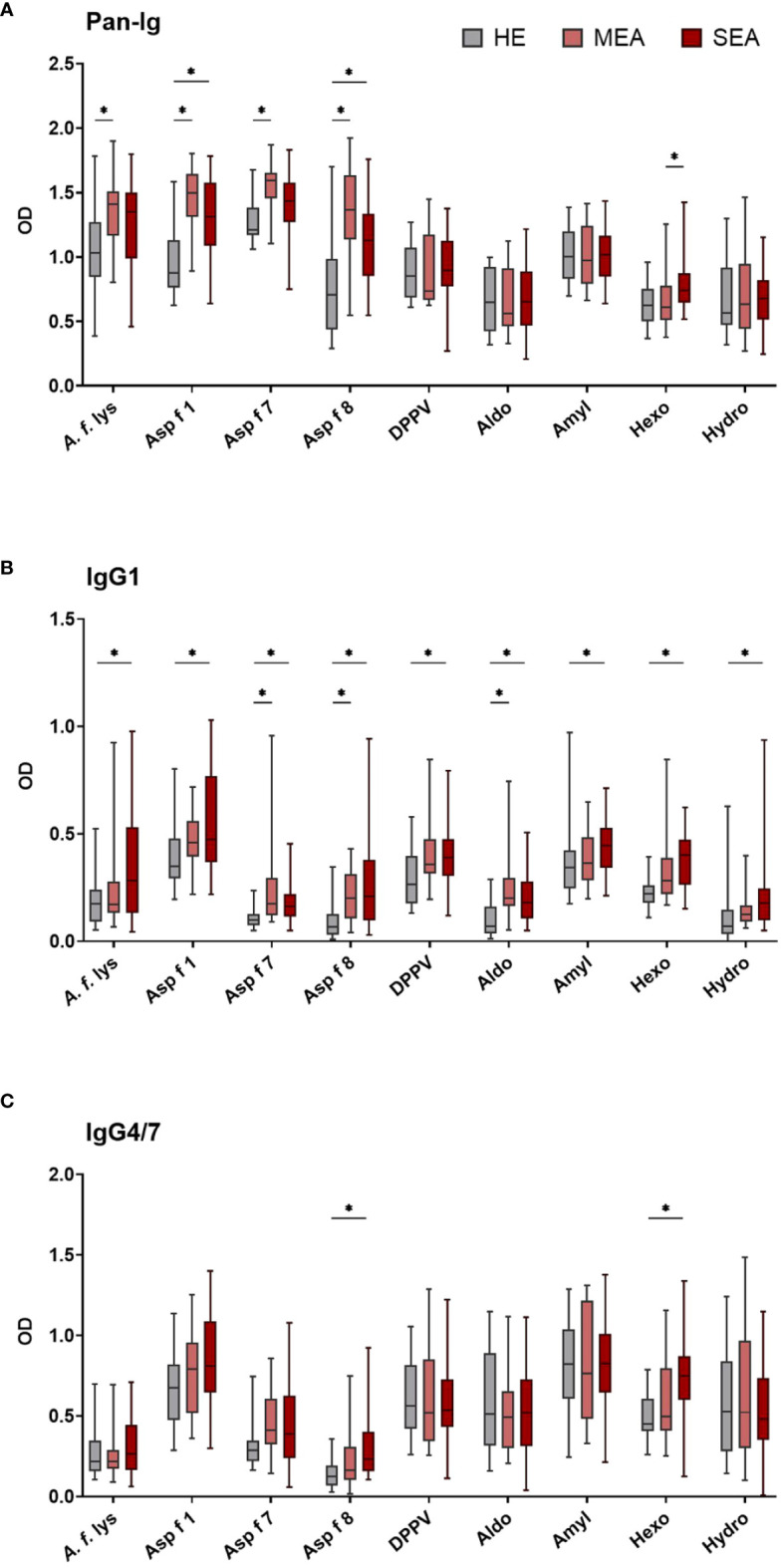
Serum IgG1 binding to *A. fumigatus* antigens is elevated in EA. *A. fumigatus* antigen-binding serum Ig was determined by ELISA. Plates were coated with *A. f.* lys or *r* antigens (Asp f 1, Asp f 7, Asp f 8, DPPV, Aldo, Amyl, Hexo, and Hydro). Serum Ig binding of all isotypes [**(A)** Pan-Ig, with polyclonal detection] and separate isotypes [**(B)** IgG1 and **(C)** IgG4/7] was quantified and compared between healthy horses (HE, *n* = 18), horses with mild-moderate asthma (MEA, *n* = 20), and horses with severe equine asthma (SEA, *n* = 24). Blank-reduced optical densities (ODs) are shown as box plots. Significant differences according to Mann–Whitney tests (*p >* 0.05) are indicated by asterisks (*). Comparisons between different antigens or isotypes’ OD do not allow direct deduction or comparison of Ig concentrations.

Serum antibody binding to different *A. fumigatus* antigens by all immunoglobulins (Pan-Ig) and the isotypes IgM, IgG1, IgG3/5, IgG4/7, IgG6, IgE, and IgA was quantified by ELISA. Serum from MEA or SEA yielded increased Ig binding to several or all *A. fumigatus* antigens, respectively (**Figure 1** shows the isotypes with group differences detected). Serum Ig binding patterns were usually similar for the different antigens and *A. f.* lys.

Compared to serum from HE, MEA yielded increased Pan-Ig binding to four *A. fumigatus* antigens (*A. f.* lys, Asp f 1, Asp f 7, and Asp f 8, **Figure 1A**), and serum from SEA had increased Pan-Ig binding to two of these antigens (Asp f 1 and Asp f 8, **Figure 1A**). Moreover, the only group difference between MEA and SEA within the study was found in higher serum Pan-Ig binding to Hexo in SEA than MEA (**Figure 1A**). IgG1 binding to three antigens (Asp f 7, Asp f 8, and Aldo) was increased in MEA compared to HE and to all antigens in SEA compared to serum from HE (**Figure 1B**). IgG4/7 binding to two antigens (Asp f 8 and Hexo) was higher in serum from SEA than HE (**Figure 1C**), but similar between MEA and HE, or between MEA and SEA.

Serum IgM, IgG3/5, IgG6, IgE, and IgA binding to all *A. fumigatus* antigens tested was similar between the groups (**Supplementary Figure 2**). High interindividual variabilities including several samples without Ig binding detection were noted for IgG6 and IgE (**Supplementary Figures 2C, D**) and very low IgE binding (OD mainly <0.5) was observed (**Supplementary Figure 2D**).

### 3.2 BALF IgA and IgG1 binding to *A. fumigatus* antigens is elevated in EA

**Figure 2:**
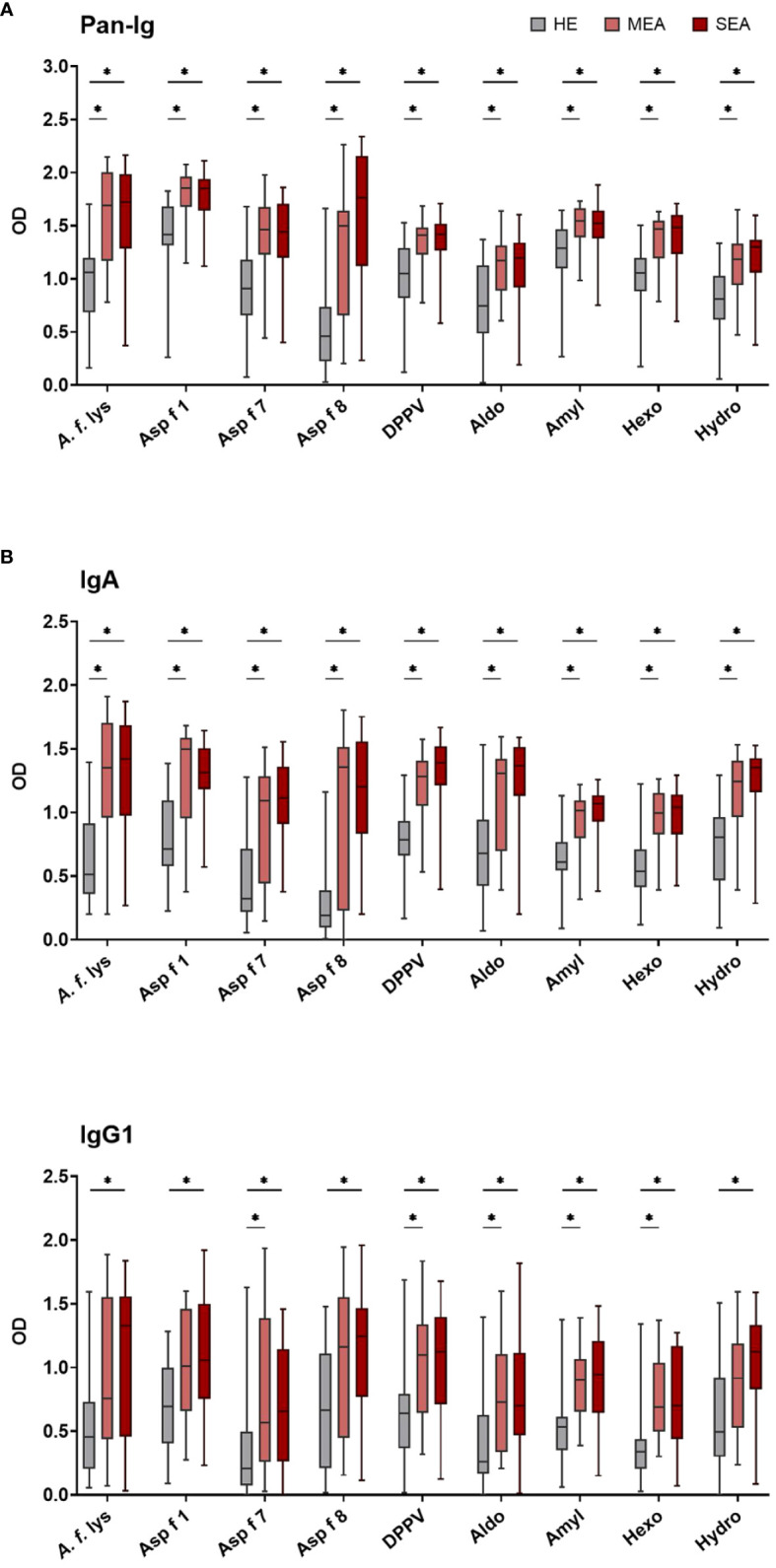
BALF IgA and IgG1 binding to *A. fumigatus* is elevated in EA. BALF Ig binding to *A. fumigatus* antigens was evaluated by ELISA. Plates were coated with *A. f*. lys or *r* antigens (Asp f 1, Asp f 7, Asp f 8, DPPV, Aldo, Amyl, Hexo, and Hydro). Binding of all Ig [**(A)** Pan-Ig] and separate isotypes [**(B)** IgA and **(C)**] IgG1) was quantified and compared between BALF from healthy horses (HE, *n* = 18), horses with mild-moderate asthma (MEA, *n* = 20), and horses with severe equine asthma (SEA, *n* = 24). Blank-reduced optical densities (ODs) are shown as box plots. Significant differences according to Mann–Whitney tests (*p >* 0.05) are indicated by asterisks (*). Comparisons between different antigens or isotypes’ OD do not allow direct deduction or comparison of Ig concentrations.

BALF from HE, MEA, and SEA horses was compared to analyze local (lung) Ig responses in EA. BALF antibody binding to *A. f.* lys and different *A. fumigatus r* proteins used as antigens was tested by ELISA for all immunoglobulins (Pan-Ig) and the isotypes IgG1, IgG3/5, IgG4/7, IgG6, IgE, and IgA. In general, BALF from MEA and SEA showed increased anti-*A. fumigatus* Ig with similar binding to the different antigens (**Figure 2** shows the isotypes with group differences detected), resulting in less heterogeneous patterns than in serum (**Figure 1**). BALF Pan-Ig and IgA binding to all *A. fumigatus* antigens was higher in MEA and SEA compared to HE (**Figures 2A, B**). BALF IgG1 binding to five antigens was higher in MEA than HE, and BALF IgG1 binding to all *A. fumigatus* antigens was higher in SEA than HE (**Figure 2C**). BALF Ig binding in MEA compared to SEA was not statistically significantly different for any isotype–antigen combination.

BALF IgG3/5, IgG4/7, IgG6, and IgE binding to all *A. fumigatus* antigens was similar between the groups (**Supplementary Figure 3**). High interindividual variabilities including several samples without Ig binding detection were noted for IgG6 and IgE (**Supplementary Figures 3C, D**) and very low BALF IgE antigen binding (OD < 0.5) was usually observed (**Supplementary Figure 3D**).

### 3.3 Total Ig contents in serum and BALF

**Figure 3:**
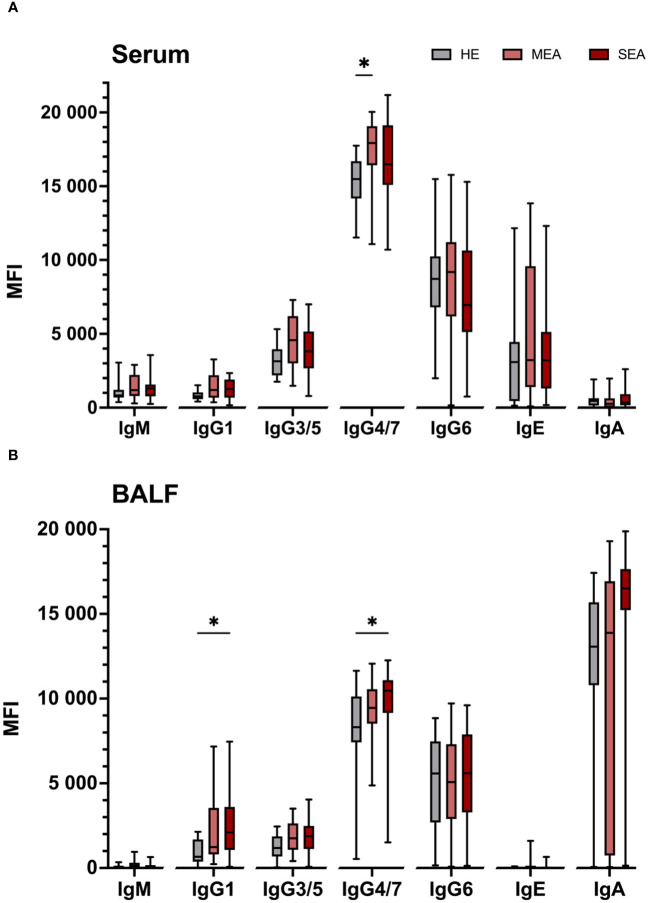
Total IgG isotypes are elevated in serum from MEA and BALF from SEA. Total Ig isotypes were quantified by bead-based assays in **(A)** serum and **(B)** BALF from healthy (HE, *n* = 18) horses or those with mild-moderate asthma (MEA, *n* = 20) or severe equine asthma (SEA, *n* = 24). Serum samples were diluted 1:50,000 for IgM and IgG, and 1:10 for IgE and IgA quantification. BALF samples were assayed undiluted. Median fluorescent intensities (MFI) are shown as box plots. Group differences according to Mann–Whitney tests are indicated with asterisks if *p >* 0.05. Comparisons between different isotypes’ MFI do not allow direct deduction or comparison of Ig concentrations.

To compare if antigen binding differences were mirrored by different total contents of the Ig isotypes, the total Ig contents were quantified by bead-based assays (**Figure 3**). Total Ig contents in serum were dominated by IgG isotypes (**Figure 3A**). Serum from MEA contained more IgG4/7 (*p =* 0.004) and tended to contain more IgG3/5 (*p =* 0.078) than serum from HE (**Figure 3A**). Median serum IgG1 contents tended to be higher in MEA and SEA than HE, albeit there was no significant difference between the groups (*p =* 0.18 or 0.16, respectively). All other serum Ig isotype contents were similar in SEA, MEA, and HE (**Figure 3A**). Serum IgE concentrations were variable between individuals (median 13.1 µg/mL; range 0.7–230.6 µg/mL), but similar between the groups (medians: HE 11.9 µg/mL; MEA 12.4 µg/mL; SEA 12.3 µg/mL).

BALF contained mainly IgA, but all IgG isotypes were detected in most samples, while IgM and IgE contents were low or undetectable in BALF (**Figure 3B**). IgE concentrations in the naïve BALF samples ranged from undetectable to 415 ng/mL (median 16 ng IgE/mL BALF) and were similar in the three groups (medians: HE 3 ng/mL; MEA 28 ng/mL; and SEA 16 ng/mL). The IgG1 and IgG4/7 contents in BALF from SEA exceeded those of BALF from HE (*p =* 0.008 and 0.019, respectively, **Figure 3B**). Albeit not significant, a trend towards higher median total BALF IgA contents in SEA compared to HE was observed (*p =* 0.12, **Figure 3B**). Median total BALF IgG1 contents tended to be higher in MEA than HE, but were not significantly different (*p =* 0.113) and all Ig isotype contents of BALF from MEA were not significantly different from HE or SEA.

Associations between isotypes were evaluated in correlations over all 62 individuals, without group stratification (**Supplementary Figure 4**). Most isotype contents did not correlate between serum and BALF, but IgA contents correlated weakly (Spearman *r* = 0.65, **Supplementary Figures 4A, B, E**). Total serum IgG1 contents correlated with total serum IgM and IgG3/5 (Spearman *r* > 0.7, *p >* 0.05; **Supplementary Figure 4A**) and weakly with IgG4/7 (Spearman *r* = 0.66, **Supplementary Figure 4A**). Total contents of IgG1, IgG3/5, and IgG4/7 in BALF all correlated with each other (all Spearman *r* > 0.7, *p >* 0.05; **Supplementary Figure 4A**), but not with total IgA or IgE in BALF (**Supplementary Figure 4A**).

### 3.4 Differentiation of EA according to Ig isotype patterns

**Figure 4:**
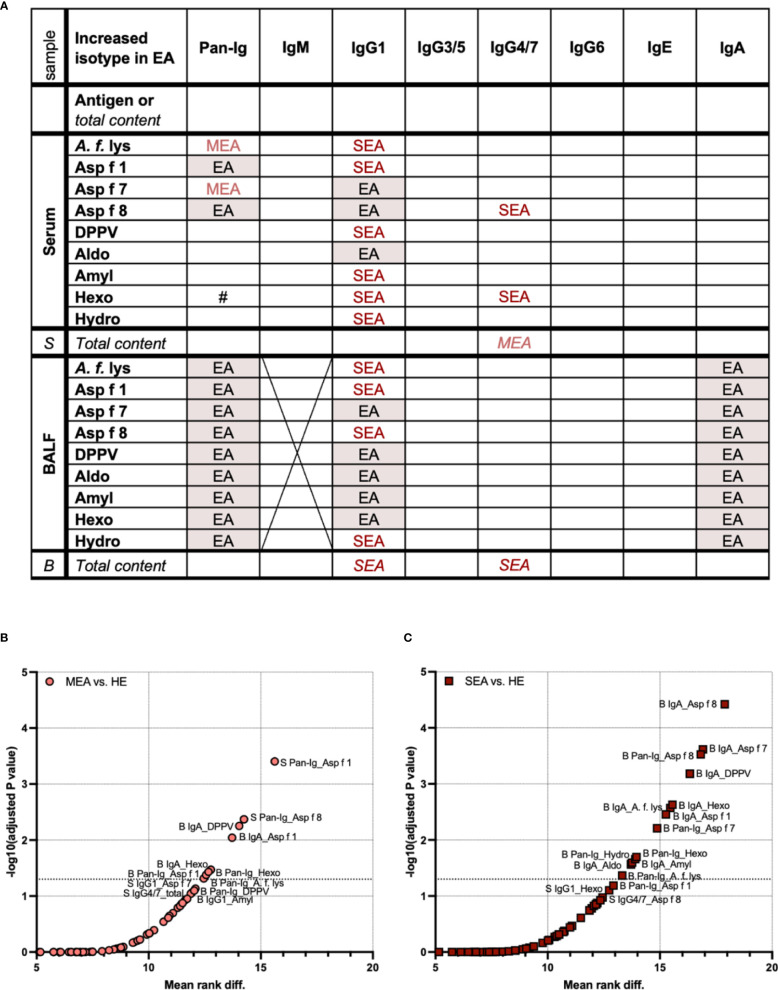
Serum and BALF Ig isotype binding patterns to *A. fumigatus* antigens to differentiate healthy and asthmatic horses. Binding of all antibodies (Pan-Ig) and six Ig isotypes to *A. fumigatus* antigens (*A. f.* lys, *r* Asp f 1, Asp f 7, Asp f 8, DPPV, Aldo, Amyl, Hexo, and Hydro) and total Ig contents in BALF (B) and serum (S) were quantified and compared between healthy horses (HE), horses with mild-moderate asthma (MEA), and horses with severe equine asthma (SEA). **(A)** An overview table summarizes group differences in all parameters analyzed. Groups with significantly higher *A. fumigatus* Ig binding or total Ig contents (in italics) are indicated; EA with red shading: MEA and SEA > HE, MEA (light red): MEA > HE, SEA (dark red): SEA > HE, #: SEA > MEA. *A. fumigatus* binding IgM in BALF was not tested due to low total IgM contents. IgG1 and IgA yielded most group differences between SEA and/or MEA and HE. Compared to serum, BALF presented with a more consistent pattern of increased antigen-binding Ig. **(B, C)** To identify the antigen–isotype combinations that best differentiate asthmatic from healthy horses according to their antibody responses to *A. fumigatus*, multiple Mann–Whitney tests were performed over all parameters (antigen-binding Ig and total Ig contents) for **(B)** MEA vs. HE, and **(C)** SEA vs. HE. Volcano plots show mean rank differences and negative logarithms of *p*-values, with annotations for significant comparisons and trends with *p* < 0.12. Additional dotted horizontal line indicates −log10 (adjusted *p*-value) = 1.3, i.e., *p* = 0.05. Differences between antigen–isotype combinations were small. Total Ig isotype contents did not yield significant differences (all *p >* 0.05). MEA vs. SEA did not yield significant differences and is not shown (all *p* > 0.3).

To deduct characteristic patterns, all Ig isotypes were compared regarding *A. fumigatus* binding and total Ig content in serum and BALF (**Figure 4**). Antigen-binding IgG1 was elevated in serum from MEA and/or SEA (EA: both higher than HE), but total serum IgG1 contents were similar between all groups. In contrast, serum IgG4/7 binding to two antigens was increased in SEA, but total serum IgG4/7 was increased in MEA compared to HE (**Figure 4A**). In addition, serum Ig binding to *A. fumigatus* antigens did not correlate with total Ig contents in serum (**Supplementary Figure 4**).

BALF antigen-binding IgG1 and total BALF IgG1 contents were elevated in SEA compared to HE (**Figure 4A**). MEA showed increased antigen-binding IgG1 for some antigens in BALF, but total IgG1 contents were not elevated (**Figure 4A**). Antigen-binding IgA in BALF, but not the total IgA content, was also increased in SEA compared to HE (**Figure 4A**). In contrast, antigen-binding IgG4/7 in BALF was not different between the groups, but total IgG4/7 content was higher in BALF from SEA compared to HE (**Figure 4A**).

Total contents of IgG1 in BALF correlated with BALF IgG1 binding to *A. fumigatus* antigens (Spearman *r* > 0.7, **Supplementary Figures 4C, G, H**), but those of IgG4/7 or IgA binding did not correlate with the total contents of these isotypes in BALF (**Supplementary Figures 4B, F**).

To test if increased antigen-binding Ig was merely explained by higher Ig contents, e.g., for BALF IgG1, Ig isotype–antigen binding was normalized to the respective Ig isotype content (ratios). Yet, increased Ig contents alone did not explain increased *A. fumigatus* antigen binding in EA. IgA binding normalized to total IgA contents was still higher in MEA or SEA than in HE regarding BALF IgA binding to all antigens (**Supplementary Figure 5A**). Normalized serum and BALF IgG1 antigen binding were still higher in EA than in HE, but the increased BALF IgG1 binding to six antigens in SEA over HE did not reach statistical significance anymore (*p =* 0.061, **Supplementary Figure 5D**). Serum IgG4/7 binding to Asp f 8 and Hexo was increased in SEA with (**Supplementary Figure 5E**) or without normalization (compare **Figure 1C**).

The matching pattern of increased antigen-binding IgG1 in EA in both serum and BALF (**Figure 4A**) could indicate a direct association of this IgG1 between both compartments and potentially a common source. To investigate this, the correlation of each antigen–isotype combination between serum and BALF was analyzed (**Supplementary Figure 4**). Antigen-binding IgG1 hardly correlated between serum and BALF (**Supplementary Figure 4C**), indicating a separation between the compartments.

Nevertheless, most group comparisons between HE and MEA or SEA yieded similar patterns over all antigens, particularly regarding BALF Ig binding (**Figure 4A**). To test if any *A. fumigatus* antigen would be representative for all selected ones or groups of antigens, we further analyzed if Ig binding correlated between different antigens (**Supplementary Figure 4**). Serum Ig binding between antigens did not correlate in general, except for that within serum IgA to most *r* antigens, but not *A. f.* lys (**Supplementary Figure 4B**). In contrast, BALF Pan-Ig, IgA, and IgG1 binding correlated between most *A. fumigatus* antigens for each isotype (**Supplementary Figure 4**). This correlation was weak between BALF IgG1 binding to Asp f 8 and BALF IgG1 binding to the insoluble antigens tested here (**Supplementary Figure 4C**).

To narrow down if single antigen–isotype combinations were most robust to differentiate immune responses in EA, these were considered if their comparisons’ *p*-values still indicated significance after correction for *all* multiple comparisons, including those of antigen-binding Ig and total Ig contents in serum and BALF (**Figures 4B, C**). Consistent with the separate analyses, these overall isotype patterns differentiated SEA from HE more clearly (higher −log10 *p*) and for more antigen–isotype combinations (13/135 combinations, **Figure 4C**) than MEA from HE (8/135 combinations, **Figure 4B**). Only increases of antigen-binding Ig, but not total contents, of MEA vs. HE or SEA vs. HE were significant over all comparisons. Yet, total IgG4/7 content in serum yielded a trend (*p =* 0.087) to differentiate MEA from HE (**Figures 4B, C**). Ig binding to *A. fumigatus* did not differentiate MEA and SEA well (all multiplicity corrected *p >* 0.3, data not shown).

MEA and HE were best differentiated by serum Pan-Ig binding to Asp f 1 (lowest *p*-value and highest rank difference), followed by serum Pan-Ig binding to Asp f 8, BALF IgA binding to Asp f 1, DPPV, and Hexo, and Pan-Ig binding to *A. f.* lys, Asp f 1, and Hexo (**Figure 4B**). SEA was best differentiated from HE by BALF IgA binding to Asp f 8, followed by BALF IgA binding to *A. f.* lys, Asp f 1, Asp f 7, DPPV, Aldo, Amyl, and Hexo or BALF Pan-Ig binding to *A. f*. lys, Asp f 7, Asp f 8, Hexo, and Hydro (**Figure 4C**). Serum Ig binding was of minor importance for SEA differentiation from HE and only yielded trends of differences in serum IgG1 binding to Hexo and serum IgG4/7 binding to Asp f 8 (*p =* 0.79 and *p =* 0.11, respectively) in these multiple comparisons.

In summary, the characteristic pattern that differentiated MEA or SEA from HE was increased *A. fumigatus* antigen-binding BALF IgA and IgG1. Serum-Ig binding also differentiated EA and HE, particularly MEA and HE. Serum and BALF Ig antigen binding was not predictive of each other. Antigen-binding Ig differentiated EA and HE better than total Ig contents in BALF or serum. While differences between different antigens’ Ig binding patterns were small, some *A. fumigatus* antigens might be slightly superior to differentiate immune responses in EA from HE (**Figures 4B, C**).

## 4 Discussion

This study showed increased *A. fumigatus* binding Ig in EA compared to healthy horses (HE), matching previous descriptions in principle (18, 26, 33, 34). This finding is in concordance with indications of excessive immune responses to fungal aeroantigens in EA (4). The differences in Ig binding to fungal antigens between asthmatic and healthy horses’ BALF Ig were more distinct than those in serum, which is in agreement with previous reports that included both sample types (19, 33, 34).

In contrast to many studies that focused on IgE only (17, 19, 23, 32), we assessed several isotypes. Ig binding differences were not apparent in IgE, but IgA and IgG1 binding was significantly increased in EA. These Ig increases clearly discriminate EA from HE and point to a relevance of local (lower airway) IgA and IgG1 responses to fungal antigens in EA. IgA was the most prominent isotype of Ig responses to *A. fumigatus* in BALF, representing a local response in the lower airways as the site of EA pathology manifestation. *A. fumigatus* binding IgA in BALF was mainly increased in both MEA and SEA, agreeing with a previous report of elevated BALF IgA in SEA (33). The IgA binding pattern to all antigens was congruent with that of Pan-Ig, and IgA appears to be the main Ig isotype binding fungal protein antigens in the lower respiratory tract of horses.

Antigen-specific IgA is likely produced locally by plasma cells in the mucosa. IgA is transported across mucosal epithelia by the pIg receptor and therefore directionally secreted on mucosal surfaces (67). This is reflected in the overall dominance of total IgA in BALF and accumulation of *A. fumigatus* binding IgA in BALF. In contrast to the preferred IgA binding to fungal antigens in BALF from the lower respiratory tract here, a dominance of local specific IgG over IgA responses in the equine upper respiratory tract was observed after herpesvirus infection (38, 47). The preferred respiratory mucosal Ig isotype response might differ between lower and upper respiratory tract compartments and depend on the antigen type and chronicity of exposure.

We observed elevated *Aspergillus*-binding IgG1 systemically in serum and locally in BALF. Asthmatic horses suffering from constant or frequent fungal exposure may particularly respond with IgG1 secretion. To our knowledge, this has not been described in EA yet and the current report is the first to analyze this equine sub-isotype separately in BALF. IgG1 was the main EA-differentiating isotype that bound to *A. fumigatus* in serum or BALF, even though IgG1 was not the main total Ig isotype in either sample. Elevated serum IgG1 (formerly termed IgGa) binding to Asp f 7 or BALF IgG binding to *A. fumigatus* lysate was also reported in previous studies on SEA (18, 34).

IgG1 is secreted in horses’ immune responses to many antigens like bacterial toxins, viral antigens, or parasitic allergens, and is characterized by fast induction in nasal secretions and serum after infection or vaccination (38, 40, 43, 46, 47). Moreover, equine IgG1 declines fast after clearance of infection, and has a short estimated serum half-life of 17 days (38, 47, 65). Accordingly, IgG1 could indicate elevated responses to recent or chronic exposure to *A. fumigatus* antigens in EA. These responses may not be properly regulated in asthmatic horses and, therefore, their secreted anti-*Aspergillus* IgG1 increases in comparison to healthy horses with similar exposure. Nevertheless, immune horses (protected from clinical disease) challenged with herpesvirus (EHV-1) intranasally do not respond with IgG1 and lack local inflammation, while susceptible horses first mount an IgG1 response and local pro-inflammatory cytokine responses (38, 47). Equine IgG1 can accordingly be interpreted to indicate an adaptive response in inflammatory contexts. Additionally, and in contrast to IgA, equine IgG1 can activate complement C1 and bind to Fc receptors to induce oxidative burst (39). These effector functions could contribute to neutrophilic inflammation, hypersecretion, tissue damage, and increased oxidative stress in EA (4, 31).

Increased human serum IgA, IgG1, and IgG2 against *A. umbrosus* lysate were similarly reported in hypersensitivity pneumonitis (alveolitis and farmer’s lung), which is based on IgG-mediated type III hypersensitivity occurring in humans chronically exposed to barn dust and other irritants (15, 68). Furthermore, anti-commensal IgG was shown to contribute to intestinal inflammation in ulcerative colitis in humans and a mouse model, including excessive Th17 responses and neutrophilic inflammation (69). The association of neutrophilic inflammation, increased antigen-binding IgG, and type 3 responses in mouse models matches the findings of increased IgG in EA shown here and previously (18, 26, 33) and the Th17 increase in SEA described in other studies (4, 28, 29).

Total Ig analysis was conducted to determine if the observed increase in *A. fumigatus* antigen-binding Ig may solely be due to a general increase in Ig secretion, or *vice versa*. *A. fumigatus*-binding IgG1 and total IgG1 correlated in BALF, but antigen binding of other IgG was not merely reflective of total IgG contents in the samples. Even if *Aspergillus* antigen-binding IgG1 was normalized to total IgG1 in BALF, increased binding in EA was still observed in MEA or SEA compared to HE. In contrast to IgG1, BALF IgG4/7 antigen binding was not increased, but total BALF IgG4/7 in SEA was. This suggests that antigens other than *A. fumigatus* provoke increased total BALF IgG4/7 in SEA and increased total serum IgG4/7 in MEA. *A. fumigatus* antigen-binding IgG1 and IgA isotypes seem to be specifically provoked and their increases in MEA and SEA support the relevance of these responses to fungal antigens in EA.

Given the association of equine IgG4/7 with type 1, and IgG3/5 and IgE with type 2 immune responses in horses (38), elevated total BALF IgG1 and IgG4/7 in SEA rather point to non-type 2 responses locally in the lower respiratory tract. Horses with MEA seemed to react similar in principle, but their Ig isotype pattern in BALF was less distinct from HE. Elevated total serum IgG in MEA may indicate a systemic inflammatory response with a tendency of increased type 1 associated IgG4/7. However, this polarization was not very clear regarding the total serum Ig isotype contents and was not reflected in *Aspergillus-*binding Ig in MEA. Additionally, the previously reported serum IgG3/5 bias in SEA for *A. fumigatus* antigen binding on immunoblots (24, 70) could not be corroborated in this study, which included a larger and different cohort of horses. The previous study on horses with SEA compared to HE analyzed serum Ig binding to *A. fumigatus* on immunoblots (24) and different methods likely contribute to the different results compared to ELISA analyses here.

Finally, our current data do not support a complete divergence of, e.g., type 2 responses to fungal antigens in asthma in contrast to non-type 2 in healthy horses, but rather indicate the same type of Ig responses (local IgA and systemic IgG) that are regulated in HE but not EA. This dysregulated Ig production could result in the increase of several IgG isotypes in EA and the correlation between the main IgG subtypes observed in BALF or serum.

BALF IgE binding was not increased in EA in our study applying naïve BALF in ELISA. This result contrasts a previous approach, which employed concentrated BALF and the use of microarrays and also included Asp f 8 (19). However, even serum IgE binding to *A. fumigatus* antigens, which was evaluated and reported as increased in EA in several studies (18, 19, 23, 26), was not different in asthmatic horses compared to healthy horses here. IgE binding was not detected at all in many serum samples of either group. Moreover, total IgE contents did not differ systematically either. In other studies, horses were matched based on environmental factors and sampling times (19, 32), which was not possible for our present study. IgE is affected by several factors, such as season and parasite burden, which were not controlled here and could have impacted the results (38). In addition, elevated Asp f 7-binding serum IgE detected by ELISA was reported in one family of 56 horses with SEA, but not another (65 horses), which points to an influence of the genetic background on IgE responses in EA (18). Effects on serum IgE binding to Asp f 7 and Asp f 8 by genetic predisposition and environment were likewise reported in 448 Lipizzan horses, without distinction of EA (71). The horses included in our current study were of diverse breeds and genetic backgrounds, which is reflective of the typical EA patient population in Germany and matches the lack of a distinct breed predisposition for EA (2, 72). Environmental risk factors for EA (dry hay exposure, straw, and predominant indoor husbandry) were present for most horses in this study, in all three groups analyzed, but with a lower proportion of horses with reported straw exposure and SEA (**Supplementary Table 2**) (9).

Differences in IgA and IgG binding in this study were very clear without matching of the samples and suggest that these major isotypes in BALF or serum will likely also compete with IgE binding to the antigens *in vitro*, hampering IgE detection in the ELISA (27, 40). Total IgG increases in BALF or serum were not directly congruent with IgG binding in this study and did not distinguish HE and EA as well as Ig binding to *A. fumigatus* antigens. Therefore, the quantification of Ig isotype contents is interesting for a comprehensive characterization of Ig responses in EA, but not sufficient for its analysis, and the analysis of antigen-specific responses is necessary to understand EA pathogenesis.

A clear preference to use single allergens over crude extract in serology has been determined for equine *Culicoides* hypersensitivity, but was not indicated here for EA using eight selected *A. fumigatus* antigens (25). The different *A. fumigatus* antigens tested here were mainly similar to each other regarding their immunogenicity. This was contrary to the hypothesis of single major antigens or allergens determining the immune response. It is also in contrast to the increased IgE binding to Asp f 8 that distinguished HE and SEA in previous analyses better than other allergens or *Aspergillus* extracts in panels of up to 153 extracts and 231 pure proteins on microarrays (19, 22, 26). However, immune responses to fungi can include multiple antigens of similar importance (73). Broad sensitization has also been described for ABPA in humans, yet specificity is improved by the use of selected *r* allergens for serology (51).

The *r* antigens included here were all selected based on previous indications of their relevance as immunogens in EA (17, 24, 26, 34, 37). However, their advantage over *A. fumigatus* lysate as an antigen source for IgG and IgA serology was small in this study. It is possible that IgA- and IgG-inducing *A. fumigatus* antigens detected in this approach are more diverse than IgE-inducing allergens (25, 73). A lack of dominating *A. fumigatus* antigens in EA would complicate antigen-specific therapeutic strategies as well as diagnostics. To improve comparability between different studies, purified *r* antigens facilitate better standardization than mixtures like lysates with immanent batch-to-batch variability and usually undetermined contents of each allergen and antigen (23, 25, 51).

Small differences in the Ig binding patterns in the present study might inform the preferable use of some of the *A. fumigatus* antigens for EA serology. Comparing all antigen–isotype combinations, the antigens previously described in the context of EA, Asp f 8 and Asp f 7, distinguished SEA and HE well regarding BALF IgA and Pan-Ig binding (18, 19, 23, 26). The allergens Asp f 1 and DPPV were similar in this respect and the newly investigated antigens, e.g., Hexo, as well as the *A. fumigatus* lysate were only slightly inferior. Serum Pan-Ig binding to Asp f 1 or Asp f 8, and BALF IgA binding to DPPV, Asp f 1, and Hexo distinguished MEA and HE. The utility of BALF for SEA and MEA distinction from HE supports using BALF as the diagnostic sample of choice to further analyze individual horses’ immune response to these specific antigens. If only serum is available, the distinction is not as powerful, but some antigen–isotype combinations might still be informative to describe the antibody response against *A. fumigatus* in EA.

Many of the *A. fumigatus* antigens used here have homologous proteins in other fungi, which are common in hay dust and barn environments. Proteins with over 90% similarity to each allergen and antigen included here have been described in several other *Aspergillus* spp., like *Aspergillus ugadawae*, *Aspergillus felis*, and *Aspergillus lentulus*, which all encode a 60S acidic ribosomal protein P2 with >90% identity to Asp f 8 (UniProt consortium, Q9UUZ6). With our analysis focused on one mold species, it is not clear if *A. fumigatus* particularly or several fungi have provoked the Ig binding responses to the antigens observed. Increased serum Ig binding to yeast antigens was also demonstrated in SEA before (70). Such shared or homologous antigens may pose a challenge to identify the specificity of Ig responses in EA but could also constitute an opportunity for broadly applicable serological diagnostic tools or immune-therapeutic approaches in the future.

Cross-reactivity might explain the observed overall similar Ig binding patterns of BALF Ig, which usually correlated between the antigens. If susceptible horses inhale several fungi including different *Aspergillus* spp., this might result in a broader immune response and similar Ig binding patterns towards fungal antigens in general. Serum IgG binding did not usually correlate between all antigens matching the less global Ig binding increase in serum compared to BALF in EA.

Qualitative differences of *A. fumigatus* antigen binding or total Ig were not detected between MEA and SEA in the present study. The two EA phenotypes were usually similar to each other, while both groups had increased Ig compared to HE. MEA yielded serum IgG1 binding differences to HE for fewer antigens than SEA, and IgG4/7 binding to two antigens was only increased in SEA vs. HE. BALF IgG1 binding was also only significantly elevated to 5/9 antigens in MEA, but to all analyzed *A. fumigatus* antigens in SEA. Overlap in single clinical parameters between horses with MEA and SEA was likewise observed and is immanent to the classification as suggested in the current consensus that considers a combination of parameters (1, 2). Horses with clear dyspnea and neutrophilic BALF cytology are easily diagnosed as SEA, while healthy horses must be confirmed by normal BALF cytology in the absence of clinical signs. All other horses, if not excluded, were classified as MEA here, rendering this group already phenotypically heterogeneous with a range from subclinical, mild EA only identified by abnormal BALF cytology or increased tracheal mucus, to moderate EA with clinical signs but not dyspnea at rest, and moderate neutrophilic cytology or other BALF cytology alterations (e.g., increased mast cells or eosinophils). Most horses with MEA and SEA included here showed neutrophil-dominated BALF cytology but quantitative differences in clinical severity and the degree of the BALF neutrophilia. It is therefore possible that most horses in both groups represented the same endotype of EA in this study, but different severity or stages of the same disease. This would match the mainly similar Ig patterns between MEA and SEA. Differences in IgG1 and IgG4/7 could also be attributed to the intensity and duration of provocation, which could be investigated further in environmentally controlled trials and longitudinal observations. The latter approach could also clarify the impact of the (i) duration of the disease and (ii) the timing of sampling in relation to environmental provocation with, e.g., hay dust. Here, only sampling at one time point was possible, and development of Ig responses in EA could not be observed longitudinally.

## 5 Conclusions

This study analyzed binding of Ig isotypes to several *A. fumigatus* antigens and compared them between serum and BALF of healthy horses, horses with MEA, and those with SEA. Our comprehensive analysis revealed local IgA as well as local and systemic IgG1 as hallmarks and potential targets of further studies of antigen-specific Ig.

All included antigens contributed to *A. fumigatus* immunogenicity. Therefore, the theory of single major antigens advancing immune response in EA could not be confirmed.

*A. fumigatus* Ig binding in MEA compared to SEA was mostly similar. This might point to neutrophilic MEA and SEA as different stages of the same endotype. With regard to IgG1, MEA presented a less consistent increase in *A. fumigatus* binding Ig than SEA, and it remains to be clarified mechanistically if IgG1 is a cause or result of increasing severity. Total Ig concentrations also showed differences between horse groups, but only BALF IgG1 content reflected the pattern of the isotype’s *A. fumigatus* binding in SEA. Increased *A. fumigatus* binding IgG1 could lead to increased total IgG1 contents or *vice versa*. In the other isotypes, however, total contents likely depend considerably on Ig specific for antigens other than *A. fumigatus*.

The relevance of IgE and a T2 endotype or IgE-mediated allergy hypothesis for EA pathogenesis is not supported by our current data. Certainly, BALF IgA and IgG1 antigen binding informs a new perspective on EA pathogenesis, and their dynamics could be analyzed further in longitudinal studies.

## Data availability statement

The original contributions presented in the study are included in the article/**Supplementary Material**. Further inquiries can be directed to the corresponding author.

## Ethics statement

The animal studies were approved by Landesdirektion Sachsen, Germany. The studies were conducted in accordance with the local legislation and institutional requirements. Written informed consent was obtained from the owners for the participation of their animals in this study.

## Author contributions

MJ: Writing – review & editing, Writing – original draft, Visualization, Methodology, Investigation, Formal analysis, Data curation, Conceptualization. AK: Writing – review & editing, Writing – original draft, Visualization, Methodology, Investigation, Formal analysis, Data curation, Conceptualization. BW: Writing – review & editing, Validation, Resources, Methodology, Investigation, Formal analysis. CR: Writing – review & editing, Resources, Methodology. SL: Writing – review & editing, Investigation, Formal analysis, Data curation. MK: Writing – review & editing, Investigation, Formal analysis, Data curation. CA: Writing – review & editing, Resources, Investigation, Formal analysis, Data curation. KL: Writing – review & editing, Supervision, Resources, Methodology, Investigation, Formal analysis, Conceptualization. CS: Writing – review & editing, Writing – original draft, Visualization, Validation, Supervision, Resources, Project administration, Methodology, Investigation, Funding acquisition, Formal analysis, Data curation, Conceptualization.
