# Supplementary material for: Aspergillus fumigatus binding IgA and IgG1 are increased in bronchoalveolar lavage fluid of horses with neutrophilic asthma
Source: Front Immunol. 2024 Jun 17;15:1406794. doi: 10.3389/fimmu.2024.1406794 (PMC11215007; doi:10.3389/fimmu.2024.1406794)
Supplement: Supplementary file 1 [file DataSheet_1.pdf]

1 Supplementary Figures

A

Abbreviation	Protein name	Uniprot	IPTG concentration	Expression time
DPPV [1]	Dipeptidylpeptidase V	B0XRV0	0.3 mM	2 h 30 min
Aldo	Class II aldolase/adducin domain protein	B0XWR5	1 mM	3 h
Amyl	Glucoamylase	B0XSV7	2 mM	4 h
Hexo	Beta-hexosaminidase	B0Y9W3	0.5 mM	4 h
Hydro	Peptide hydrolase	B0XX53	1 mM	3 h

[1] described as allergen

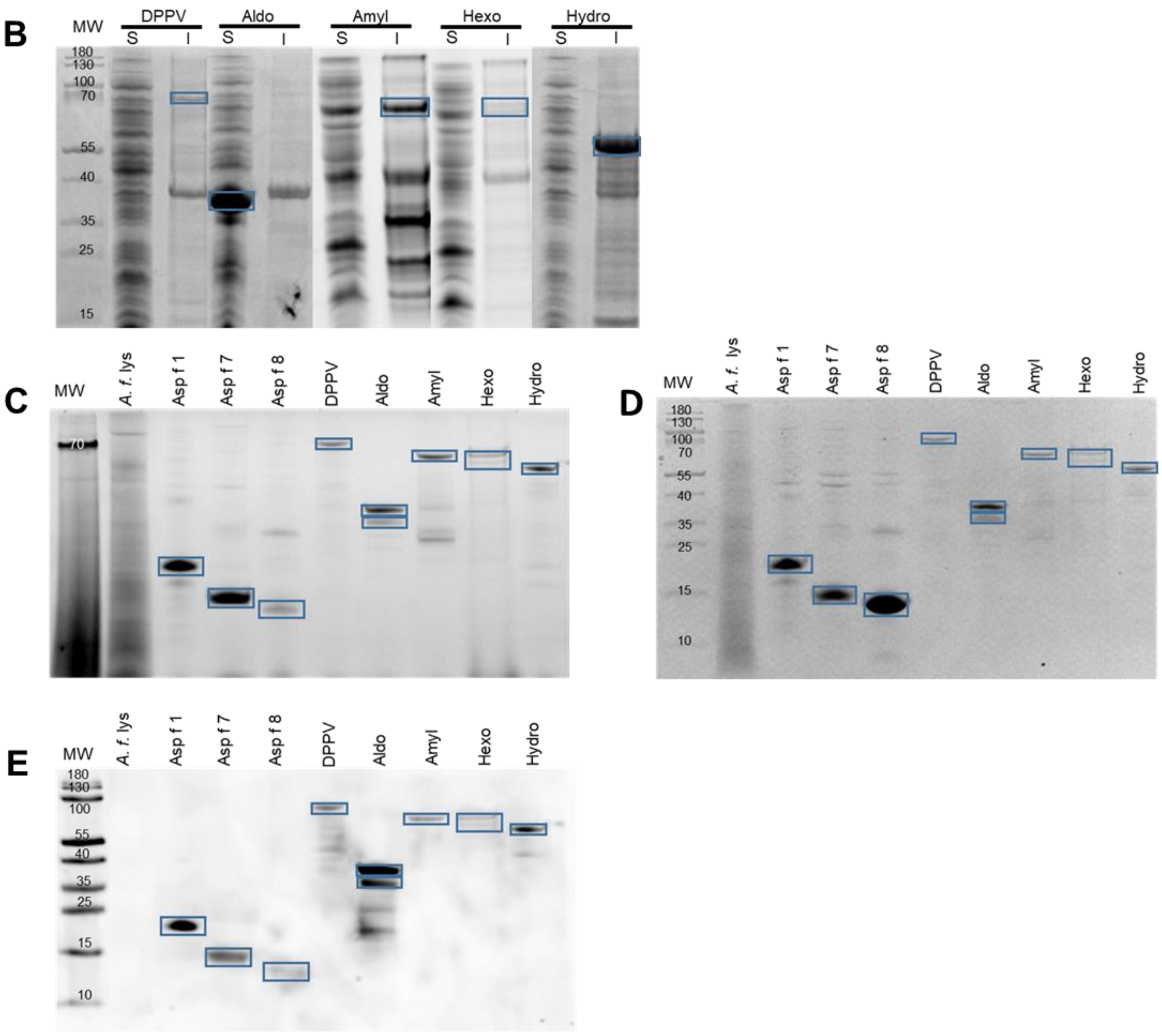

Supplementary Figure 1 ***A. fumigatus*** **antigen characterization**

Supplementary Figure 1 *A. fumigatus* antigen characterization
(A) Expression of five new *r* antigens in *E. coli* was optimized and the expression conditions depending on the antigen of interest are noted (IPTG isopropyl-ß-D-thiogalactopyranoside). (B) Solubilities of five new *r* antigens in *E. coli* lysates were assessed on SDS-PAGE according to antigen detection in the soluble (S, PBS) or insoluble (I, urea buffer) fraction. Coomassie stained proteins in SDS-gels are shown with blue rectangles highlighting the antigens at their molecular weight (MW, marker in first lane): DPPV dipeptidyl-peptidase V 80 kDa; I, Aldo class ll aldolase/ adducin domain protein 32 kDa, S; Amyl glucoamylase 67 kDa, I, Hexo beta-hexosaminidase 67 kDa, I; Hydro peptide hydrolase B0XX53 54 kDa, I. (C/D) *A. fumigatus* lysate (*A. f.* lys) and eight purified *r A. fumigatus* antigens were separated by SDS-PAGE and visualized with tryptophan fluorescence/Coomassie staining: Previously described allergens, Asp f 1 (20 kDa), Asp f 7 (27 kDa), and Asp f 8 (11 kDa), and those purified and described here (A) are highlighted with blue rectangles and were identified at their expected molecular weights. (E) His tags were detected on a corresponding immunoblot from tryptophan fluorescence gel (C) with anti-His-tag antibody followed by goat-anti-mouse AlexaFluor® 647 (visualized as Cy5 fluorescence). Aldo and Hexo contained degradation products, which yielded His tag detection in addition to the protein band of the expected size. Note that the (red) 70 kDa band of the protein ladder (MW) is visible in tryptophan fluorescence (C) while (blue) 180–10 kDa bands are visible in Cy5 (E).

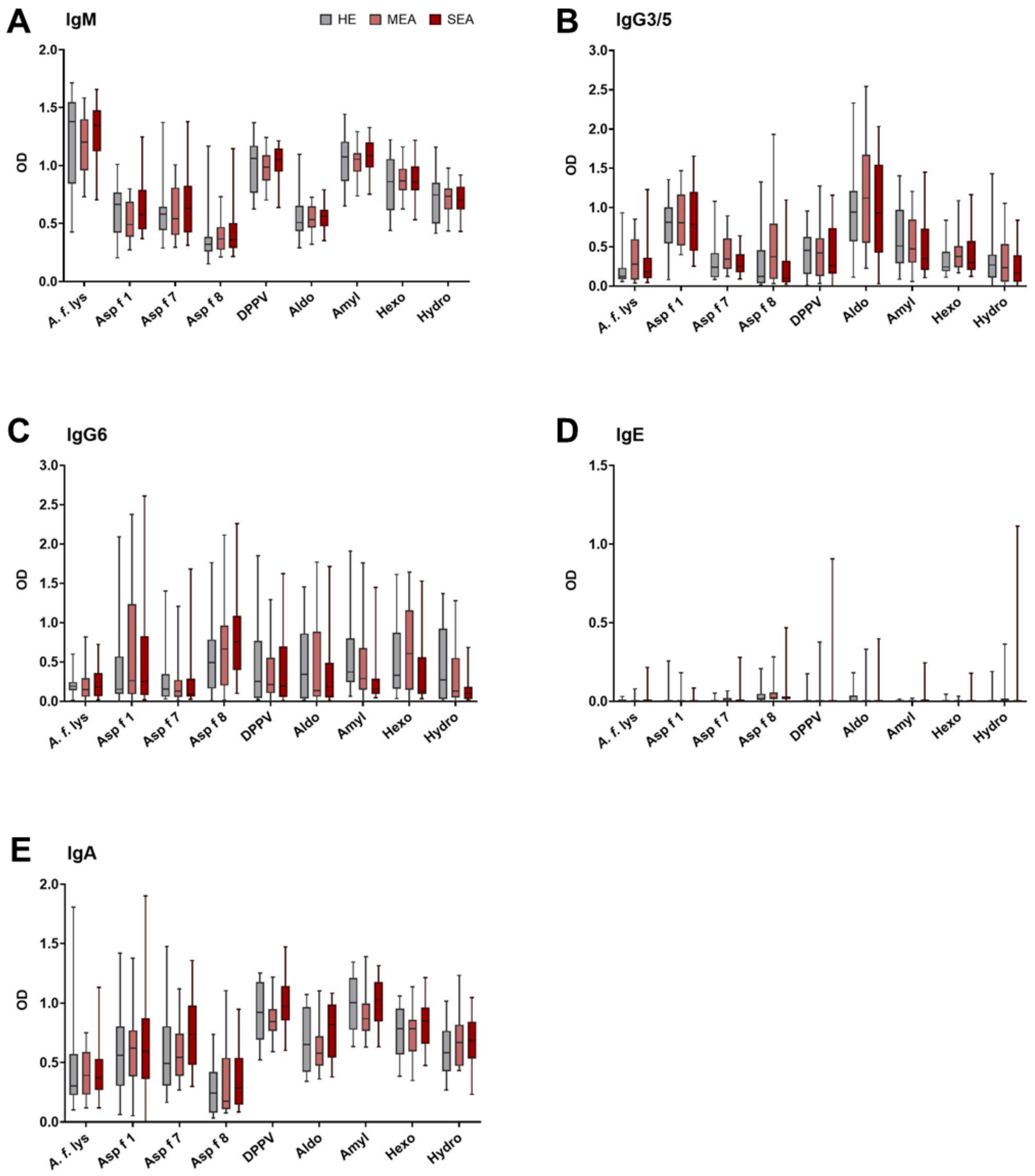

Supplementary Figure 2 Serum Ig binding of many isotypes to *A. fumigatus* antigens was similar between groups.

Serum Ig binding to *A. fumigatus* antigens was determined by ELISA. Plates were coated with *A. f.* lys and *r* antigens (Asp f, 1, Asp f 7, Asp f 8, DPPV, Aldo, Amyl, Hexo, and Hydro). Ig binding was quantified and compared between serum from healthy horses (HE, n=18), horses with mild-moderate (MEA, n=20), and horses with severe equine asthma (SEA, n=24). Similar serum Ig binding to antigens between all groups was detected for IgM (A), IgG3/5 (B), IgG6 (C), IgE (D), and IgA (E). Comparisons were performed by Mann-Whitney tests of blank-reduced optical densities (OD) and all yielded $p>0.05$. Comparisons between different antigens or isotypes' OD do *not* allow direct deduction or comparison of Ig concentrations.

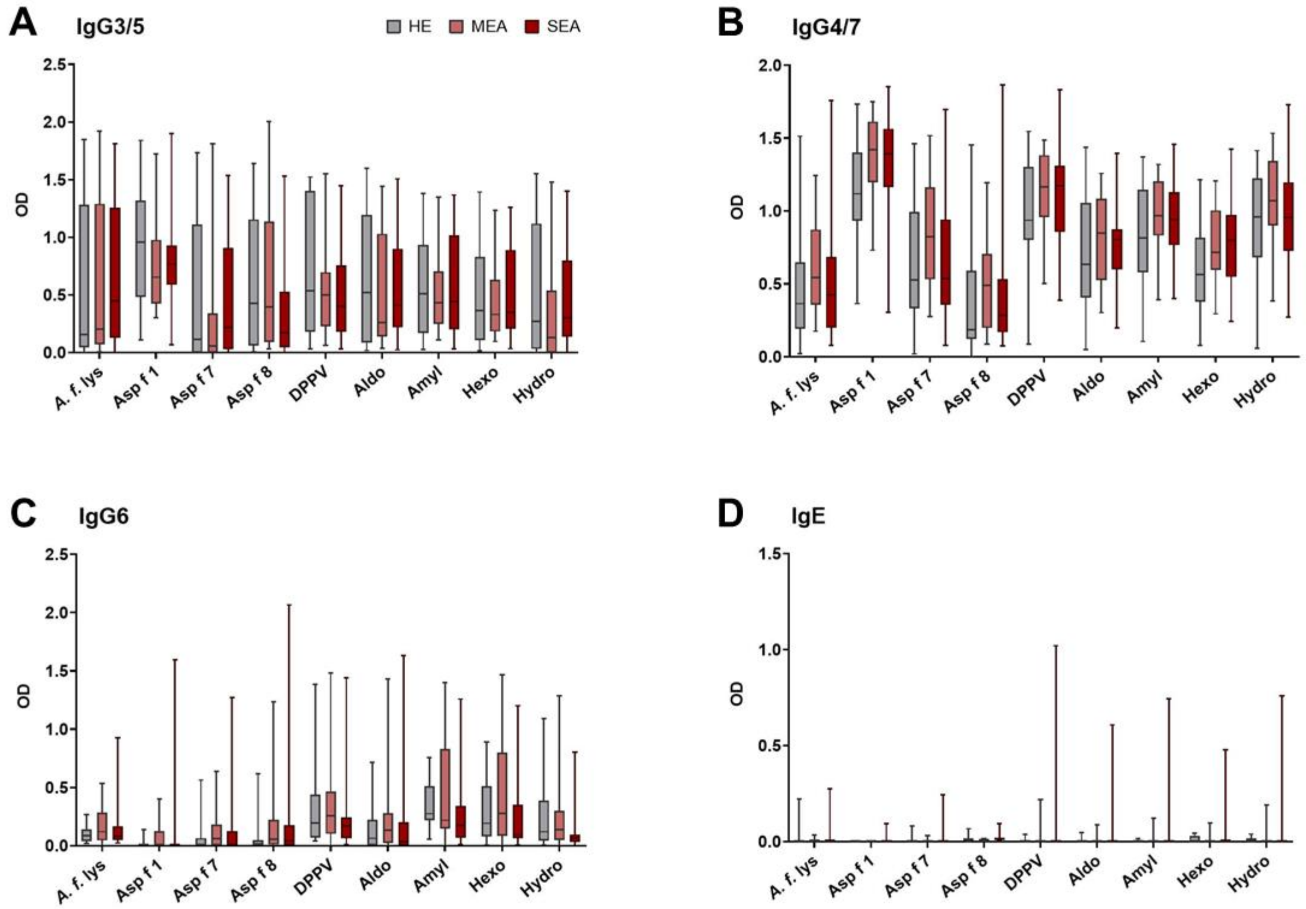

Supplementary Figure 3 BALF Ig binding of many isotypes to *A. fumigatus* antigens was similar between groups.

BALF Ig binding to *A. fumigatus* antigens was determined by ELISA. Plates were coated with *A. f.* lys and *r* antigens (Asp f, 1, Asp f 7, Asp f 8, DPPV, Aldo, Amyl, Hexo, and Hydro). Ig binding was quantified and compared between BALF from healthy horses (HE, n=18), horses with mild-moderate (MEA, n=20), and horses with severe equine asthma (SEA, n=24). Similar BALF Ig binding to the antigens in between the groups was detected for IgG3/5 (A), IgG4/7 (B), IgG6 (C), and IgE (D). Comparisons were performed by Mann-Whitney tests of blank-reduced optical densities (OD) and all yielded p>0.05. Comparisons between different antigens or isotypes' OD do *not* allow direct deduction or comparison of Ig concentrations.

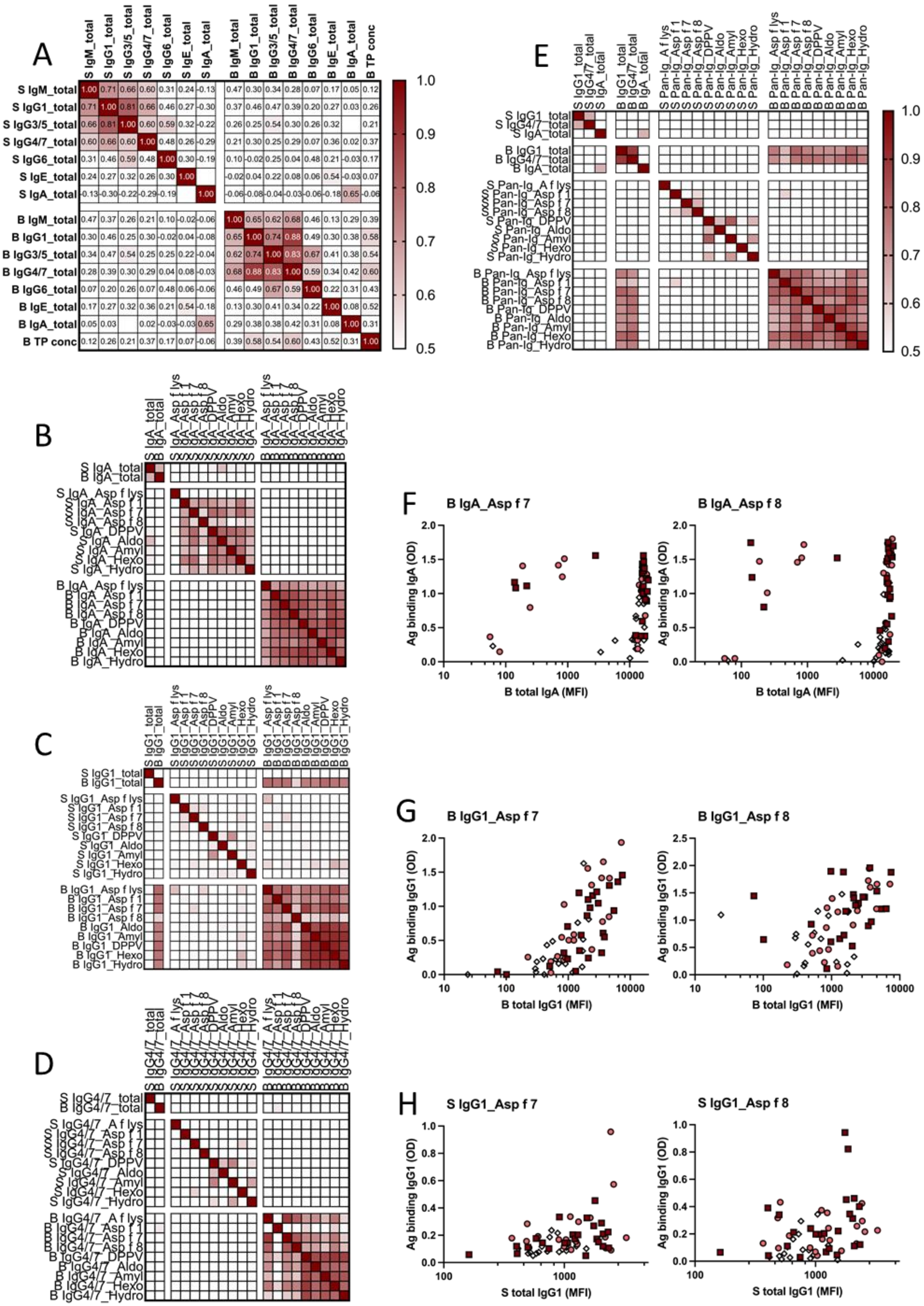
A
B
C
D
E
F
G
H
B IgA_Asp f 7
B IgA_Asp f 8
Ag binding IgA (OD)
B total IgA (MFI)
B IgG1_Asp f 7
B IgG1_Asp f 8
Ag binding IgG1 (OD)
B total IgG1 (MFI)
S IgG1_Asp f 7
S IgG1_Asp f 8
S total IgG1 (MFI)

Supplementary Figure 4 Correlation of total Ig isotype contents and Ig binding of different *A. fumigatus* antigens.

Correlations between the total Ig isotype contents and Ig binding in serum and BALF of all 62 individual horses without group separation were analyzed by Spearman correlation and are highlighted in red shading in a heat-map if Spearman $r>0.5$ and $p<0.05$. (A) total Ig content associations (B) IgA, (C) IgG1, (D) IgG4/7, (E) Pan-Ig binding associations. Correlation examples are plotted for Asp f 7 and Asp f 8 binding (F) BALF IgA vs total IgA (Spearman r= 0.28 and 0.34), (G) BALF IgG1 vs total IgG1 (Spearman r= 0.78 and 0.58), and (H) serum IgG1 vs total IgG1 (Spearman r= 0.40 and 0.37). HE as open diamonds, MEA as light red filled circles, SEA as dark red filled squares.

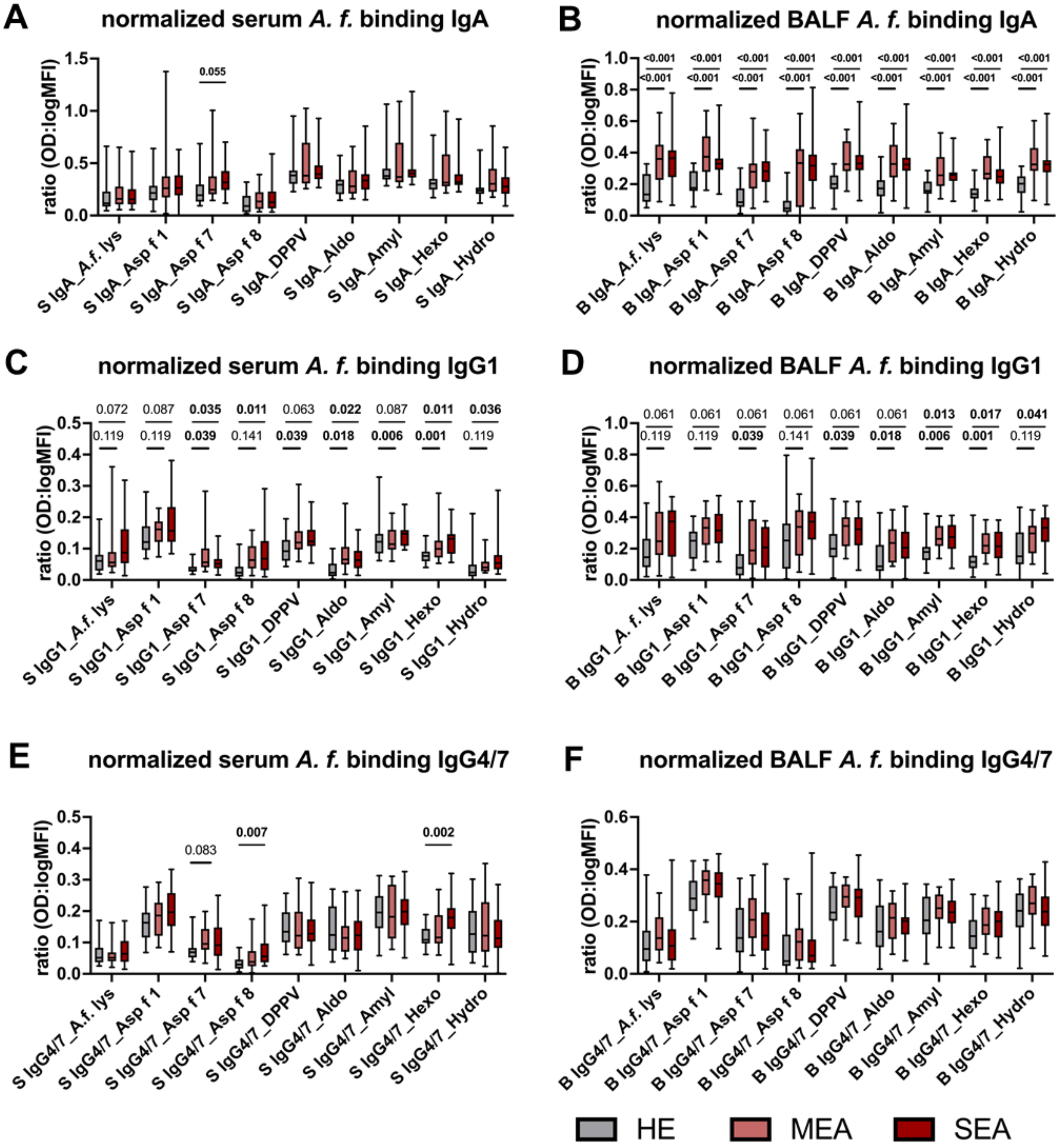

Supplementary Figure 5 Ig binding of *A. fumigatus* antigens is not merely dependent on total Ig contents.

To control for different total Ig isotype contents (MFI from bead-based assays) in serum or BALF of healthy and asthmatic horses, Ig isotype binding to *A. fumigatus* antigens (OD from ELISA) was normalized as ratios of blank-reduced OD: logarithmized blank-reduced MFI for each serum and BALF Ig isotype in the respective samples. (A, B) IgA, (B, C) IgG1, and (D, E) IgG4/7 are plotted as box plots. Group differences in Mann-Whitney tests are indicated by *p*-values (if $p < 0.15$). Significantly different comparisons ($p < 0.05$) in bold. Most significant increases of serum and BALF IgG1 binding in MEA or SEA vs. HE (compare Figures 1 and 2) were still detected after normalization, while some *p*-values were higher after normalization. Normalized IgG1 binding did not appear significantly higher in SEA anymore, but other comparisons became statistically significant if normalized IgG1 binding was assessed. The group comparisons of increased BALF IgA binding to all antigens in MEA and SEA and the increased serum IgG4/7 binding to Asp f 8 and Hexo in SEA were unaffected by normalization to their total Ig contents.

2 Supplementary Tables

Supplementary Table 1
Target gene amplifications of *A. fumigatus* proteins with primer sequences and restriction site.

Abbreviation	Protein name of target gene	Uniprot	Forward primer (restriction site) [2, 3]	Reverse primer (restriction site) [3, 4]
DPPV [1]	Dipeptidylpeptidase V	B0XRV0	*CGGCATCATATGGGAGCTTTCCGCTGGCT* [2]	*GCAATAGCGGCCGCGTTATAATTCACAACCGGGACAA* [3]
Aldo	Class II aldolase/adducin domain protein	B0XWR5	*CGCGTTCATATGCGTGCTTCTTTCATTCTCTTCTC* [2]	*GTATATGCGGCCGCCCAGCCGCTCTCCAAGC* [3]
Amyl	Glucoamylase	B0XSV7	*GCTTTACATATGGCTCCTCAGTTATCCGCTC* [2]	*CGTTCACTCGAGCCGCCAAGTATCATTCTCGG* [4]
Hexo	Beta-hexosaminidase	B0Y9W3	*AATGTTGCGGCCGCATGCTCATCTCCAGCATCTGC* [3]	*GCTTTACTCGAGCGCGACGGCACTTTGGTC* [4]
Hydro	Peptide hydrolase	B0XX53	*CGCTCACATATGGTCACCATGAAGCTGCTCTAC* [2]	*GCTTTACTCGAGCTGCTCAACCCGGTCCTTG* [4]

[1] described as allergen, restriction sites for (underlined): [2] Ndel, [3] Notl [4] Xhol

Supplementary Table 2 Environmental risk factors for asthma in horses

Group	HE	MEA	SEA	all
Data available from n (%)	16/18 (89%)	14/21 (67%)	19/23 (83%)	49/62 (79%)
Risk factors				
Dry hay exposure [1]	88%	92%	89%	90%
Straw exposure [1]	86%	83%	44%	69%
Mainly housed indoors [2]	60%	62%	56%	59%

[1] hay and straw exposure may be direct, from feed or bedding of the horse examined, or indirect, by feed or bedding of neighbouring horses.

[2] horses usually kept indoors for > 12h per day

Supplementary Table 3 Clinical score evaluated for healthy horses and horses with mild to moderate or severe equine asthma (1).

**Parameter**	**Description**	**Range**	Ranges in<br>[1] Lavoie et al., 2019 (2), or<br>[2] Woodrow et al., 2022 (3)
**Nasal discharge (ND)**	0 no ND or serous<br>1 mild mucous ND<br>2 moderate mucopurulent ND<br>3 severe mucopurulent ND	0–3	[1, 2] 0–3
**Nasal flare (NF)**	0 physiological normal nostril movement<br>1 inspiratory NF<br>2 inspiratory and expiratory NF, visible movement of the nostrils<br>3 inspiratory and expiratory NF, no movement of the nostrils	0–3	[1, 2] 0–1
**Cough**	0 none or 1x provoked after tracheal compression<br>1 several provoked coughs, by tracheal compression or after re-breathing<br>2 spontaneous cough<br>3 coughing attack lasting > 30 seconds	0–3	[1, 2] 0–3
**Respiratory Rate**	0 <16/minute<br>1 16-20/minute<br>2 21-30/minute<br>3 >30/minute	0–3	[1] 0–4<br>[2] 0–3
**Abdominal lift**	0 normal abdominal effort<br>1 slight expiratory flattening of the ventral flank<br>2 obvious abdominal flattening, heave line at most extending halfway between cubital joint and *tuber coxae*<br>3 obvious abdominal lift, heave line extending beyond halfway between cubital joint and *tuber coxae*	0–3	[1, 2] 0–3
**Lung auscultation at rest**	0 normal lung sounds<br>1 increased sounds louder than physiological trachea<br>2 crackles, wheezes	0–2	[1, 2, 3] 0–6
**Lung auscultation after re-breathing**	0 physiological lung sounds<br>1 increased sounds louder than physiological trachea<br>2 crackles, wheezes	0–2	[1] not included<br>[2] 0–2 for delayed recovery or induced cough
			[1,2] also considered tracheal sounds (0–3)
**Score sum**	**Sum of all scores above**	**0–19**	**[1] 23**<br>**[2] 22**

[3] two points for each, bronchial sound, crackles, wheezes, also if observed after re-breathing

1. Gressler AE, Lübke S, Wagner B, Arnold C, Lohmann KL, Schnabel CL. Comprehensive Flow Cytometric Characterization of Bronchoalveolar Lavage Cells Indicates Comparable Phenotypes Between Asthmatic and Healthy Horses But Functional Lymphocyte Differences. *Front Immunol* (2022) 13: doi: 10.3389/fimmu.2022.896255

2. Lavoie J -P., Bullone M, Rodrigues N, Germim P, Albrecht B, Von Salis-Soglio M. Effect of different doses of inhaled ciclesonide on lung function, clinical signs related to airflow limitation and serum cortisol levels in horses with experimentally induced mild to severe airway obstruction. *Equine Vet J* (2019) 51:779–786. doi: 10.1111/evj.13093

3. Woodrow JS, Hines M, Sommardahl C, Flatland B, Lo Y, Wang Z, Sheats MK, Lennon EM. Initial investigation of molecular phenotypes of airway mast cells and cytokine profiles in equine asthma. *Front Vet Sci* (2022) 9:997139. doi: 10.3389/fvets.2022.997139
